# The genome and preliminary single-nuclei transcriptome of *Lemna minuta* reveals mechanisms of invasiveness

**DOI:** 10.1093/plphys/kiab564

**Published:** 2021-12-06

**Authors:** Bradley W Abramson, Mark Novotny, Nolan T Hartwick, Kelly Colt, Brian D Aevermann, Richard H Scheuermann, Todd P Michael

**Affiliations:** 1 The Plant Molecular and Cellular Biology Laboratory, The Salk Institute for Biological Studies, La Jolla, California 92037, USA; 2 Department of Informatics, J. Craig Venter Institute, La Jolla, California 92037, USA; 3 Department of Pathology, University of California San Diego, La Jolla, California 92093, USA; 4 Division of Vaccine Discovery, La Jolla Institute for Immunology, La Jolla, California 92037, USA

## Abstract

The ability to trace every cell in some model organisms has led to the fundamental understanding of development and cellular function. However, in plants the complexity of cell number, organ size, and developmental time makes this a challenge even in the diminutive model plant Arabidopsis (*Arabidopsis thaliana*). Duckweed, basal nongrass aquatic monocots, provide an opportunity to follow every cell of an entire plant due to their small size, reduced body plan, and fast clonal growth habit. Here we present a chromosome-resolved genome for the highly invasive Lesser Duckweed (*Lemna minuta*) and generate a preliminary cell atlas leveraging low cell coverage single-nuclei sequencing. We resolved the 360 megabase genome into 21 chromosomes, revealing a core nonredundant gene set with only the ancient *tau* whole-genome duplication shared with all monocots, and paralog expansion as a result of tandem duplications related to phytoremediation. Leveraging SMARTseq2 single-nuclei sequencing, which provided higher gene coverage yet lower cell count, we profiled 269 nuclei covering 36.9% (8,457) of the *L. minuta* transcriptome. Since molecular validation was not possible in this nonmodel plant, we leveraged gene orthology with model organism single-cell expression datasets, gene ontology, and cell trajectory analysis to define putative cell types. We found that the tissue that we computationally defined as mesophyll expressed high levels of elemental transport genes consistent with this tissue playing a role in *L. minuta* wastewater detoxification. The *L. minuta* genome and preliminary cell map provide a paradigm to decipher developmental genes and pathways for an entire plant.

## Teaser

The genome and single-nuclei transcriptome of the Lesser Duckweed *Lemna minuta* provide preliminary evidence that tandem gene duplication and tissue-specific expression play roles in invasiveness.

## Introduction

Single-cell sequencing has ushered in a new era of biology where it is possible to characterize cell types and function with unprecedented detail. In plants, this has resulted in detailed single-cell RNA-seq and Assay for Transposase-Accessible Chromatin (scATAC-seq) datasets primarily on different organ types from well-studied model organisms (Denyer et al., 2019; Ryu et al., 2019; Dorrity et al., 2021; Gujas et al., 2020; Marand et al., 2020; Song et al., 2020; Liu et al., 2020a, 2020b; Chen et al., 2021; Lopez-Anido et al., 2021; Xu et al., 2021). Most plant studies to date start with protoplast isolation, which has the potential to miss some recalcitrant cell types and often require correction to control for transcriptional changes elicited by lengthy enzyme treatment and centrifugation steps (Ryu et al., 2019; Cuperus, 2021). In addition, there are different single-cell approaches that generally either result in assaying many cells with lower gene coverage (10X Genomics) or fewer cells with deeper gene coverage (SMARTseq2; Yamawaki et al., 2021). For example, SMARTseq2 sequencing coupled with fluorescence-activated cell sorting (FACS) of nuclei, or single-nuclei RNA sequencing (snRNA-seq), provides deep transcriptome coverage of individual nuclei (Krishnaswami et al., 2016) while avoiding loss of cells or introducing issues associated with the protoplast isolation timing. The use of snRNA-seq in plants is nascent but can extend as a broader application for studying abiotic/biotic treatments and whole plant analysis (Farmer et al., 2021; Long et al., 2021; Picard et al., 2021).

**Figure 1:**
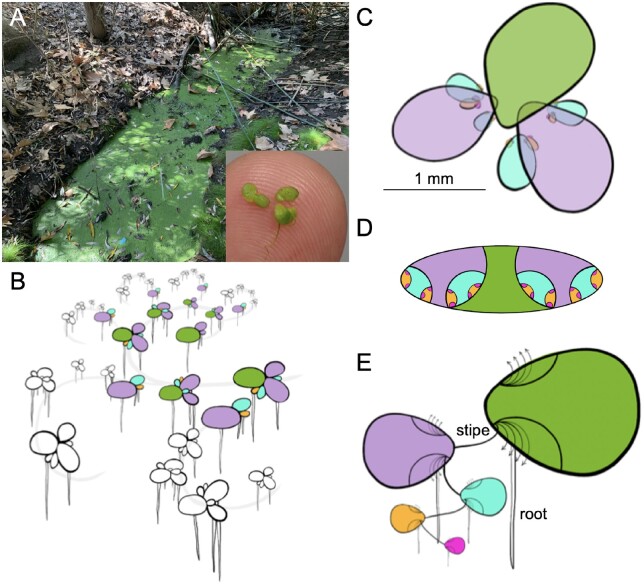
*Lemna minuta* growth highlighting its anatomical analogs to other plants. A, *L. minuta* (lm5633) growing as a dark green mat of fronds in a sewage slough at Cotton Creek Park, Encinitas, CA, USA. The inset highlights the small size (∼1 mm) of several fronds on a fingertip. B, Cartoon of the *L. minuta* generational contribution that leads to the dense frond mat: mother (M; green), daughter (D; purple), GD (blue), great grand-daughter (GGD; orange), and great, great grand-daughter (GGGD) fronds (pink). The gray line represents how they are connected through exponential growth. C, A single M frond view with the attached D fronds that have GD, GGD, and GGGD fronds nested in the two meristematic pockets. D, A “nested doll” view of one M frond and the maturing generations of D, GD, GGD, and GGGD in the pocket. E, One interpretation of the *Lemna* frond is that the M frond is an axillary stem that has two poaches or bracts where the D frond is attached by a stipe or internode. The D, GD, GGD, and GGGD progression then is similar to a branching structure of a generic plant, and the root-like structure at each subsequent axillary node is equivalent to an adventitious root. The arrows indicate that multiple internodes will emerge from the axillary stem over time (Landolt, 1986).

Most single-cell studies to date have been on specific plant organ tissue, such as the root, meristem, or inflorescence, that encompass limited cell trajectories. Duckweed, aquatic plants in the family Lemnaceae, provide a unique opportunity to follow cells through all developmental time scales from one population due to its minimal morphology and clonal pattern of growth (Figure 1). These basal monocots are some of the smallest and fastest growing plants on Earth, ranging in size from under a millimeter to centimeter, with some species doubling in under a day (Michael et al., 2020). There are five genera in the Lemnaceae with three having a flat leaf-like structure called a frond and root-like structures that may act as rudders or anchors (*Spirodela*, *Lemna*, and *Landoltia*); the other two lack roots and are spherical or flat (*Wolffia* and *Wolffiella*). Before the model plant Arabidopsis (*Arabidopsis thaliana*) rose to prominence, Duckweed were an important system for reductionist biology leading to fundamental understanding of chronobiology, flowering time, and phytohormone action (Acosta et al., 2021).

Duckweed grow primarily through an asexual budding process where a mother frond gives rise to a daughter frond, forming a dense clonal population representing all stages of development in their lifecycle (Figure 1, B–E). Despite being morphologically simple it has been hypothesized that the clonal population represents the most complex tissues found in plants (Landolt, 1986). The mother frond is an axillary shoot that gives rise to two pouches, or bracts, where the daughter frond is attached through a stipe, or internode, at the meristem and branches off in an alternating pattern similar to the growth of aerial tissue in more complex plants (Figure 1E). Every daughter frond has two developing generations nested within pouches, which start to grow when the daughter is at the 18-cell stage and these new fronds start to differentiate at the 30-cell stage (Figure 1D;Rimon and Galun, 1968). Since the average lifespan of a Duckweed plant is 30 d, and each frond is capable of generating 15–20 daughter fronds, the growth is exponential but also represents 256 (2^8^) fully formed but not expanded daughter fronds per mother frond (Figure 1B). Therefore, a Duckweed population represents a 3D look at a whole plant: developmental, cellular, and structural.

Duckweed also provides a compelling platform for genomic studies due to their core nonredundant gene set and relatively small genomes. The dawn of the genomics era yielded high-quality genomes and transcriptomes for nonmodel systems, which resulted in Duckweed emerging once again as an attractive system to tackle problems of cell biology and development (Acosta et al., 2021). The Greater Duckweed (*Spirodela polyrhiza*) was the first to be sequenced, revealing that in this 150-megabase (Mb) basal monocot genome there is a reduced complement of nonredundant protein coding genes (approximately 19,000) representing a core plant proteome (Wang et al., 2014; Michael et al., 2017). Subsequently, genomes for *Lemna minor* (Van Hoeck et al., 2015), *Spirodela intermedia* (Hoang et al., 2020), and *Wolffia australiana* (Michael et al., 2020) have been published, revealing small genomes at 472, 160, and 375 Mb, respectively. These sequenced genomes further support that Duckweed has a core plant gene set with few family expansions making them ideal for dissecting pathways and functional analysis.

Many species of Lemnaceae have dispersed widely beyond their natural ranges and are considered invasive species in their new habitats due to their rapid vegetative propagation (Moodley et al., 2016). A prime example of an alien invasive species is *Lemna minuta*, which is native throughout the temperate zones of the Americas, but has dispersed widely throughout Eurasia (Landolt, 1986). Analysis by Ceschin et al. maps the introduction of *L. minuta* in Europe to the 1950–1960s with a dispersal rate of 40–50 km year (Ceschin et al., 2018). This dispersal process involved crossing of seas to reach places such as Ireland (Lucey, 2003) and Malta (Mifsud, 2010), which is consistent with a role for bird mediated dispersal, and hence the common name Duckweed (Silva et al., 2018). However, despite *L. minuta* doubling in roughly 24 h, its growth rate does not explain its ability to outcompete native Duckweed (*L. minor*) or other aquatic plants (Van Echelpoel et al., 2016; Paolacci et al., 2018).

The small organism size (∼1 mm), fast growth rate (∼24 h), small genome size (∼360 Mb), and ability to exploit diverse environments led us to select *L. minuta* as a way to understand cell trajectories and function across an entire plant. *Lemna* *minuta* is emerging as a phytoremediation species due to its superior ability to remove various excess elements and toxins from wastewater (Ceschin et al., 2020; Fernández et al., 2020). This led us to hypothesize that the invasiveness of *L. minuta* may result from a specialized set of genes or cellular function, as it is apparently not solely due to its rapid growth rate. Therefore, we collected *L. minuta* from local wastewater (Figure 1A) in order to ensure we captured the natural diversity of a successful clone. Duckweed clones sequenced to date have all been from the Landolt Collection, which have been maintained clonally under aseptic lab conditions for 20–50 years (approximately 1,000 s of generations). We brought a single sterile clone into culture (lm5633) and generated a chromosome-resolved reference genome. Additionally, snRNA-seq was performed on a population of clonally propagating plants to understand gene expression profiles across the whole plant’s cellular developmental landscape. The genome and preliminary snRNA-seq analysis of lm5633 suggest mechanisms of invasiveness.

## Results

### *Lemna minuta* chromosome-resolved genome

**Figure 2:**
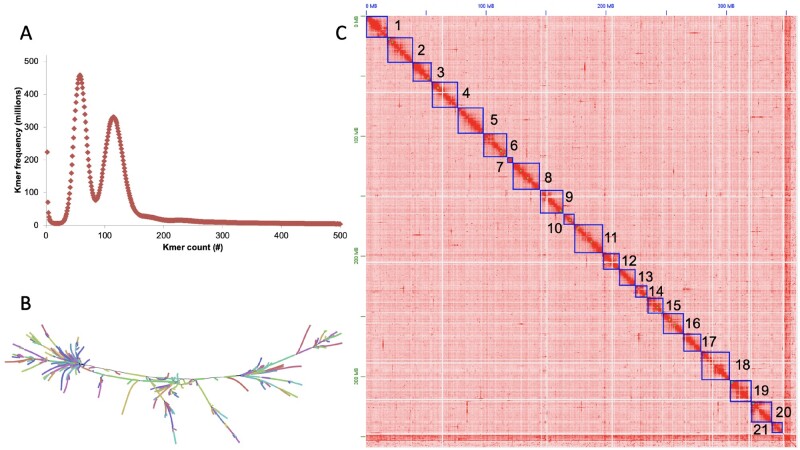
The highly heterozygous *L. minuta* (lm5633) genome resolved into 21 chromosomes. A, K-mer (*k* = 31) frequency plot for lm5633 reveals two peaks consistent with a high level of heterozygosity (2%). B, Assembly graph of a 40 Mb region visualized with Bandage shows both the heterozygous branches as well as repeat tangles, “hairballs.” C, High-throughput chromatin conformation capture (HiC) contact map resolving the polished lm5633 assembly into 21 chromosomes (darker red more contacts, lighter red to white less contacts). Chromosomes were sorted by size and renumbered before gene prediction.

*Lemna minuta* was collected from a waste water run-off in Cotton Creek Park, Encinitas, CA USA (33°2′58″N 117°17′29″W), sterilized and one clone was selected as a representative of the population for bulking; the clone ID lm5633 was assigned by the Rutgers Duckweed Stock Cooperative (RDSC; Figure 1A). We estimated the genome size as 310 Mb by k-mer (*K* = 31) frequency analysis using Illumina short reads sequencing (Figure 2A), which was smaller than the reported average genome size estimated by flow cytometry of 365 Mb (Table 1; Bog et al., 2020). The genome is highly heterozygous at 2.1% with 57% of the genome in high copy number elements (young transposable elements (TEs), centromeres, telomeres, and rDNA arrays) (Figure 2A and Table 1). We sequenced the genome using long-read Oxford Nanopore Technologies (ONT), and assembled reads into contigs using both our minimap/miniasm pipeline (Michael and VanBuren, 2020), as well as the FlyE assembler; the later assembler produced a more contiguous genome with a total length of 393 Mb, longest contig of 1.2 Mb and an N50 of 205 kb (Table 1).

**Table 1:** *Lemna minuta* (lm5633) genome statistics

Features	lm5633
Estimated genome size flow cytometry (bp)^a^	365,000,000
Estimated genome size K-mer = 31 (bp)	310,069,889
Heterozygosity prediction K-mer31 (%)	2.1
High copy repeat sequence K-mer31 (%)	57
Chromosome-resolved genome size (bp)	360,454,868
Assembled genome size (bp)	392,702,877
Contig (#)	9,473
Longest contig (bp)	1,268,926
Contig N50 length (bp)	205,076
Scaffold (#)	2,407
Longest scaffold (bp)	24,571,886
Scaffold N50 length (bp)	19,492,010
Chromosomes after HiC (#)	21
BUSCO^b^ complete (%)	72.5
Repeat predictions (%)	58.2
Telomere length average (bp)	10,085
Telomere length, longest (bp)	25,271
Predicted genes (#)	22,873

^a^ Averaged over all *L. minuta* clones tested by (Bog et al., 2020).

^b^ BUSCO library: liliopsida_odb10.

Since the genome assembly was larger than the predicted genome size, we reasoned that some of the genome was retained in two haplotypes. The genome assembly graph confirmed that excess haplotypes were retained appearing as branches in the graph, as well as suggesting a complex repeat structure (TEs) resulting in repeat tangles, or “hairballs” in the graph (Figure 2B). We assessed the completeness of the assembly with Benchmarking Universal Single-Copy Orthologs (BUSCOs; liliopsida library) finding 72.5% complete (Table 1); the lower percent complete is consistent with other high-quality chromosome resolved Duckweed genomes that are all missing the same single-copy genes in the liliopsida library (Hoang et al., 2018; Harkess et al., 2020; Michael et al., 2020). Recently, it has been shown that in novel and complex plant genomes, synteny between close relatives is a better measure of completeness than BUSCO scores (addressed below) (Zhao and Schranz, 2019).

**Figure 3:**
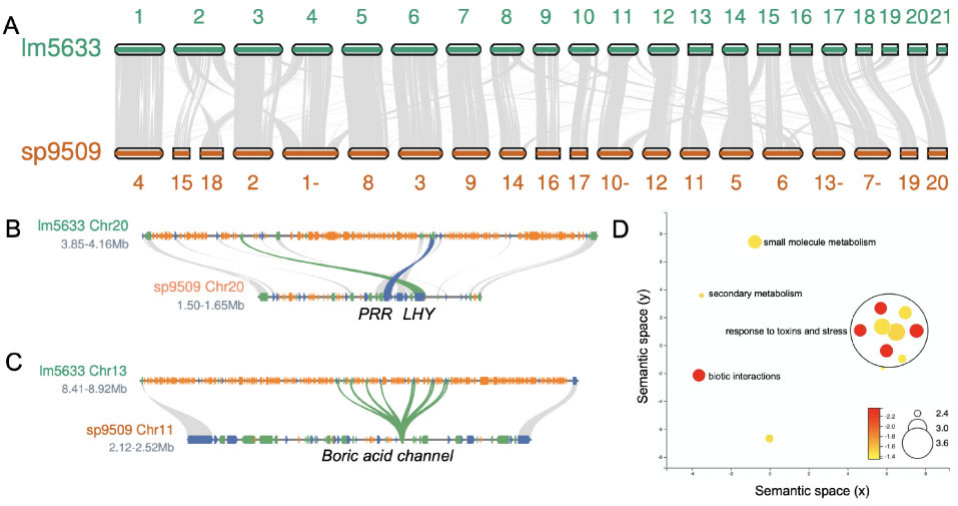
Gene family expansion in lm5633 is driven by TD. A, Lm5633 chromosomes aligned to sp9509 chromosomes with syntenic blocks (gray lines) anchoring positions between the two genomes. Chromosomes are the correct ratio between one another but are not to scale between the two species. A minus sign after the number means the chromosome has been flipped for visualization purposes. B, The single MYB transcription factor *LATE ELONGATED HYPOCOTYL* (blue line) and *PSEUDO-RESPONSE REGULATOR 7* (green line) are in tight linkage and TE fragments (orange) resulting in the region expansion in lm5633. Gray lines connect other syntenic genes (blue, forward; green reverse). C, Boric acid channel TD (green line) also shows the expansion of the lm5633 genome due to TE fragments (orange). D, multi-dimensional scaling (MDS)-based visualization of Semantic similarity between significant GO terms associated with TDs in lm5633. GO terms that are more semantically similar (shared words) will be closer together in the scatter plot. Size of the circle is the log frequency and the color (red high, and yellow low) is the log FDR.

The genome was scaffolded into 21 chromosomes using Illumina-based high-throughput chromatin conformation capture (HiC), which is consistent with published cytology (Supplemental Figure S1; Landolt, 1986). However, we noted a high level of redundant contigs in the contact map, suggesting residual haplotypes in the assembly consistent with our larger genome size and 11.8% of the *BUSCO* genes being found in duplicate (Table 1; Supplemental Table S1). We successfully purged the excess overlapping haplotypes (Supplemental Figure S2) and generated a final genome of 21 nonredundant chromosomes (Figure 2C). The final lm5633 HiC assembly was collinear with the high-quality assembly of *S. polyrhiza* clone 9509 (sp9509) chromosome structure, consistent with the lm5633 assembly completeness despite the missing *BUSCO* genes (Figure 3A).

### Lm5633 repeat characterization and gene prediction

Long read assemblies cover more repeat sequences and usually allow the identification of putative centromere sequences, definition of telomere lengths, and annotation of full-length TEs (Michael and VanBuren, 2020). Consistent with the high copy number repeat k-mer frequency estimate, we identified that 58.2% of the genome was repeat sequence, which is double that of sp9509 and similar to *W. australiana* clone 8730 (wa8730) (Table 1; Supplemental Table S2). The lm5633 genome has a gypsy/copia ratio of ∼2, similar to that found in sp9509, but distinct to wa8730 where the ratio is closer to 1 (Supplemental Table S2; Michael et al., 2017, 2020). The larger genome size of lm5633 compared to sp9509 was primarily due to the increase in retained TE fragments, which is seen in the number of repeats found intervening in the evolutionarily conserved linkage of core circadian clock genes (Figure 3B; Michael et al., 2020). Similar to *S. polyrhiza*, we did not detect high copy number centromere repeats (Michael et al., 2017), but we did identify telomere (AAACCCT) arrays that are longer (average = 10 kb; longest 25 kb) than sp9509 (average = 3 kb; longest = 6 kb), yet shorter than what we have found in wa8730 (average = 18 kb; longest = 70 kb) (Supplemental Table S3; Michael et al., 2020).

**Figure 4:**
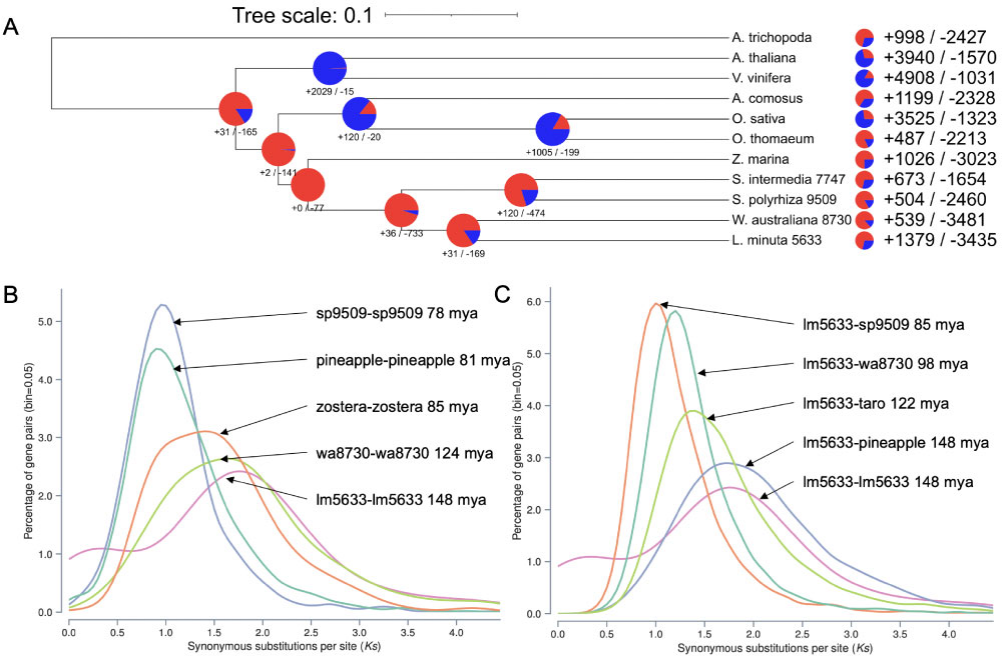
Whole genome evolution shows consistent gene family contractions in lm5633 ancestry. A, Gene family contractions (red) and expansions (blue) along the phylogenetic tree leading to minimized Duckweed genomes are shown at each node and total protein family contractions and expansions for each species (right). Lm5633 shows the greatest similarity and gene family conservation with wa8730. B, Self versus self and C, lm5633 versus other Ks plots to elucidate the WGD history of lm5633. Dating is based on the mean Ks peak for each comparison using all paralogs/orthologs. Plant species used: *A. trichopoda* (Amborella), *A. thaliana*, *Vitis vinifera* (grape), *Ananas comosus* (pineapple), *O. sativa* (rice), *Oropetium thomaeum* (oropetium), *Zostera marina* (zostera), *C. esculenta* (taro), *S. intermedia* (si7747), *S. polyrhiza* (sp9509), *W. australiana* (wa8730), and *L. minuta* (lm5633).

The sequenced Duckweed genomes have the fewest protein-coding genes found in angiosperms to date with wa8730 and sp9509 having just 14,324 and 18,507 genes, respectively (Michael et al., 2017, 2020). After masking the repeat sequence, we predicted 22,873 protein-coding genes in the lm5633 genome (Table 1), which is similar to the 22,382 and 22,245 protein-coding genes predicted in the lm5500 and si7747 assemblies, respectively (Van Hoeck et al., 2015; Hoang and Schubert, 2017). The higher gene counts for both lm5633 and si7747 compared to wa8730 and sp9509 are a result of those genomes having orthogroups with ˃10 genes, which is similar to Arabidopsis and rice (*Oryza sativa*) where gene families are much larger (Figure 4A; Supplemental Figure S3). Of the 99 lm5633 orthogroups that had ˃10 genes, 50% had between 0 and 2 genes in sp9509. The expanded lm5633 families could result from whole-genome duplication (WGD), tandem duplication (TD), proximal duplication (PD), transposed duplication, or dispersed duplication (Qiao et al., 2019). The expanded lm5633 genes were predominantly a result of TD and PD events, as compared to sp9509, leading to expansion of genes involved in pathogen defense, response to stress, and nutrient acquisition (Figure 3D; Supplemental Table S4). For instance, there is an 11 gene TD of a boric acid channel (Figure 3C), which may reflect the ability of lm5633 to extract large amounts of boron from its environment, improving survivability, and invasiveness (Fernández et al., 2020).

A comparison of orthologous proteins between lm5633 and wa8730 revealed a number of expanding orthogroups relating to the ability of lm5633 to thrive in diverse environments. We found 14 significantly expanded orthogroups in lm5633 compared to the most recent common ancestor wa8730 (Figure 4A). There are five orthogroups annotated with gene ontology (GO) terms: OG0000813, vacuolar membrane; OG0000014, signaling; OG0001260, defense response to fungus; OG0000015, detection of bacterium; OG0003377, delta14-sterol reductase activity. There are three significantly contracted orthogroups compared to wa8730 with one having an annotation for GO:0005787, signal peptidase complex. An additional expanded orthogroup, OG0000243, encodes a family of proteins relating to multidrug and toxic compound extrusion (MATE) proteins that have more than doubled from two copies in wa8730 to five copies in lm5633. The MATE transporters are often associated with increased plant resilience to toxic compounds and adaptability to metals including iron homeostasis (Upadhyay et al., 2019). These evolving orthogroups are consistent with the ability of lm5633 to thrive under adverse conditions of abiotic and biotic stress.

### Lm5633 chromosome evolution

The lm5633 genome was resolved into 21 chromosomes (Figures 2, C and 3, A), which is consistent with cytological studies (2*n* = 42; Landolt, 1986); this is one more chromosome than sp9509 and three more than si7747 (Michael et al., 2017; Hoang et al., 2020). Since *S. polyrhiza* is thought to be the basal Duckweed, we looked specifically at the synteny between the lm5633 and sp9509 chromosomes and found that 97% of lm5633 genes are in syntenic blocks of ≥1copies with sp9509, resulting in a syntenic depth of 1:1 (Supplemental Figure S4). Eighteen chromosomes share high sequence collinearity with at least five that are completely collinear, while there are several lm5633 chromosomes (2,19, and 21) that are the result of fragments from several sp9509 chromosomes (Figure 3A). However, there are no sp9509 chromosomes resulting from lm5633 fusions, consistent with the basal nature of the *S. polyrhiza* genome. We noted a similar 1:1 syntenic depth and chromosomal conservation between lm5633 and si7747 further supporting the completeness and accuracy of the lm5633 genome assembly and the conservation across Duckweed genera (Supplemental Figure S5).

It has been reported that *S. polyrhiza* has experienced at least two lineage specific WGDs (B′′/a′′) in the nongrass monocots (Wang et al., 2014; Ming et al., 2015). Consistent with this WGD history, sp9509 has a 3:1 syntenic depth compared to the *Amborella trichopoda*, which is the progenitor basal angiosperm without a WGD (Amborella Genome Project, 2013). In contrast, lm5633 has a 2:1 syntenic depth compared to *A. trichopoda*, suggesting that despite having a 1:1 syntenic depth lm5633 and sp9509 may have distinct WGD histories (Supplemental Figure S5). Therefore, we looked at the synonymous substitution (Ks) rates of lm5633 paralogs to estimate their age and found that both wa8730 and lm5633 lack a Ks peak corresponding to B”/a” WGD with only a peak corresponding the to the *tau* (τ) WGD (Figure 4B). Consistent with this, the divergence of lm5633 and pineapple coincides with the *tau* (τ) WGD shared across most of the monocot lineage (Jiao et al., 2014; Figure 4C).

There have been several conflicting studies as to whether *Spirodela* and the Duckweed lineage experienced the τ WGD (Wang et al., 2014; Ming et al., 2015; Hasing et al., 2020). It is possible that the B′′/a′′ WGD experienced by Spirodela and subsequent fractionation back to a 1:1 syntenic depth obscured the τ WGD that we see in lm5633 and wa8730. While only 4.1% of the WGD paralogs in lm5633 are found in syntenic blocks with depth greater than two, the sp9509 genome maintains almost half (49%) of its genes in blocks with syntenic depth greater than two. While most of the syntenic blocks have been fractionated back to a 1:1 syntenic depth, examples of 4:1 syntenic depth between sp9509 and lm5633 are present (Supplemental Figure S6). Additional Duckweed genomes from *Lemna*, *Landoltia*, *Wolffia*, and *Wolffiella* as well as other closely related species in the Araceae will be required to fully understand the WGD history of Duckweed.

### Predicting cell types in lm5633 with low cell coverage snRNA-seq

The unique life cycle and growth pattern of *L. minuta* plants from mother to daughter and grand-daughter (GD) fronds covers all cell developmental stages and types providing an opportunity to follow the developmental trajectory of all cells (Figure 1). We wanted to identify as many cell types as possible across the diverse developmental states with high gene coverage per cell. Therefore, we carried out snRNA-seq, which enabled the capture of an exact moment containing the cellular developmental trajectory by the immediate freezing of tissue and avoiding long protoplasting steps that can result in unsampled cell types and sample preparation artifacts (Denyer et al., 2019). We isolated individual nuclei from a population of whole lm5633 plants grown under standard laboratory conditions by FACS and then prepared libraries with SMARTseq2 to achieve high gene coverage per nuclei (Supplemental Figure S7; Supplemental Tables S5 and S6). The SMARTseq2 methodology is clearly limited by the total number of nuclei that can be captured, yet it provides the opportunity to generate higher sequencing depth per nuclei, which can facilitate a broader analysis of how specific cells are functioning. Nuclei number and transcript depth pose a clear tradeoff in snRNA-seq studies, and in this initial effort to characterize all cell types across a whole nonmodel plant, we opted for fewer nuclei with higher transcript coverage. After read and cell quality filtering, 8,457 genes or 36.9% of the annotated lm5633 genes were expressed across 269 nuclei (Supplemental Figure S7; Supplemental Figure S8). These 269 nuclei were grouped into 13 clusters based on expression profiles using all available expressed genes and not only the most highly variable genes (Supplemental Figure S7). The 269 nuclei represent an extremely small subset of all possible cells (approximately 4,000 per frond); however, this effort represents preliminary characterization of the minimal number of cells in the minimal *L. minuta* body plan and provides some direct hypotheses for in vitro validation of the predicted cell types.

**Figure 5:**
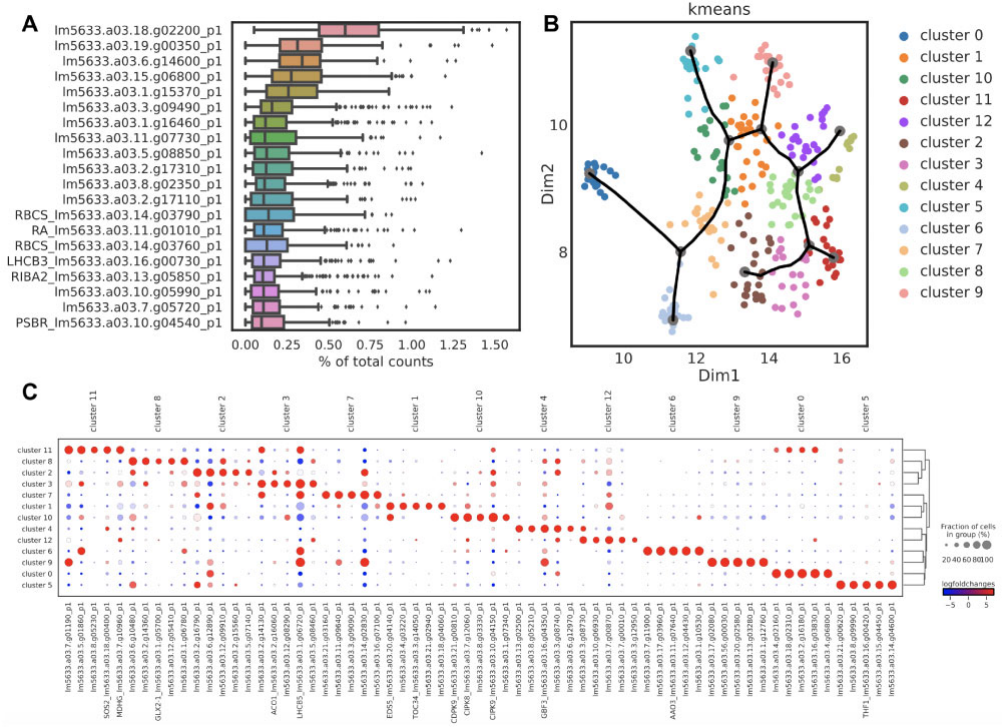
snRNA-seq of clonally propagating whole lm5633 plants. A, The most abundant transcripts reveal that highly expressed genes are generally related to photosynthesis across all cell types while some highly expressed genes lack robust annotation. The box plot describes the mean fraction of reads assigned to the 269 cells, center line, and the first and third quartiles at the left and right box limits, respectively. Additional points outside whiskers represent outlier cells. B, UMAP embedding of 13 k-means clusters in this snRNA-seq dataset. Trajectory analysis (black line) reveals a complex network where multiple branches result in putative terminally fated cell types. C, Log fold change in marker genes expressed in 269 nuclei forming 13 specific clusters shows robust separation of cell types for functional analysis. Expression level (red, high; blue, low; larger circle, more cells; smaller circles, fewer cells).

One problem encountered with nonmodel plants is the lack of robust annotation. Therefore, we employed several orthology-based methodologies to determine marker genes for the snRNA-seq clusters. For instance, only 6 of the 20 most highly expressed genes for all cells in this dataset had annotations, 5 related to photosynthesis as expected for a photosynthetic organism (Figure 5A). To functionally characterize the 13 clusters we identified marker genes for each cluster and lm5633 orthologs relative to published single-cell studies in model plants, Arabidopsis, rice, and maize (*Zea mays*; Denyer et al., 2019; Satterlee et al., 2020; Xu et al., 2021; Supplemental Table S7). We leveraged a combination of gene orthology predictions (Supplemental Table S8) of marker genes, GO annotations, and preliminary snRNA-seq trajectory analysis to define the clusters into hypothesized cell types. A gene overexpressed in a specific cluster is defined as a marker gene and in model species is usually supported by an in vivo reporter expression assay. A total of 1,733 significant candidate marker genes were determined between all 13 clusters (Figure 5B; Supplemental Figures S9 and S10; Supplemental Table S9). Of the 1,733 candidate marker genes found for the 13 clusters, we found on average each marker gene was in an orthogroup with 2.3 copies per species while 140 marker genes did not appear in any orthogroup with model plants (Supplemental Table S8). An average of 134 marker genes per cluster was determined with a total of 446 marker genes annotated based on model organism orthology, averaging to 18 annotated marker genes per cluster (Figure 5C; Supplemental Table S9).

**Figure 6:**
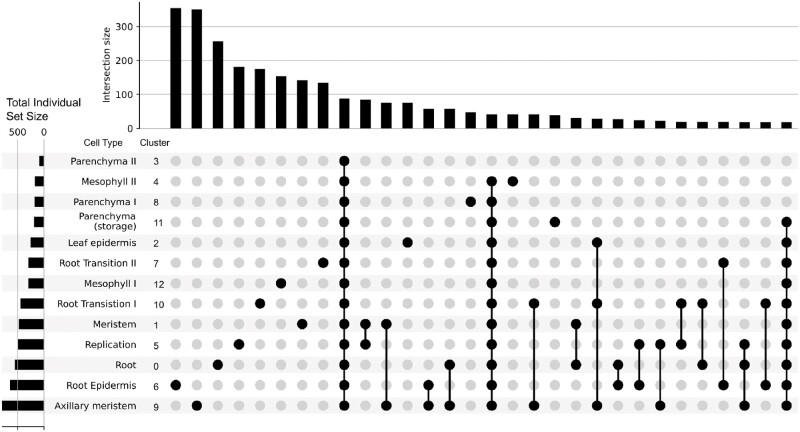
Cell-type definitions supported by GO categories. Upset plot showing GO term uniqueness associated with each cell cluster based on annotated marker genes. The bars on top count the number of GO terms unique to a specific set of clusters defined by the dots on the bottom. For example, there are 340 GO terms associated with marker genes for cell cluster 6, root epidermis, that are not associated with any marker genes for any other cell cluster. Overall, unique GO terms are associated with individual clusters (i.e. cell types) suggesting each cell type’s marker genes have a functionally unique GO annotation profile. The strongest co-occurrence of GO terms appears between the meristem–replication and meristem-axillary meristem clusters.

**Figure 7:**
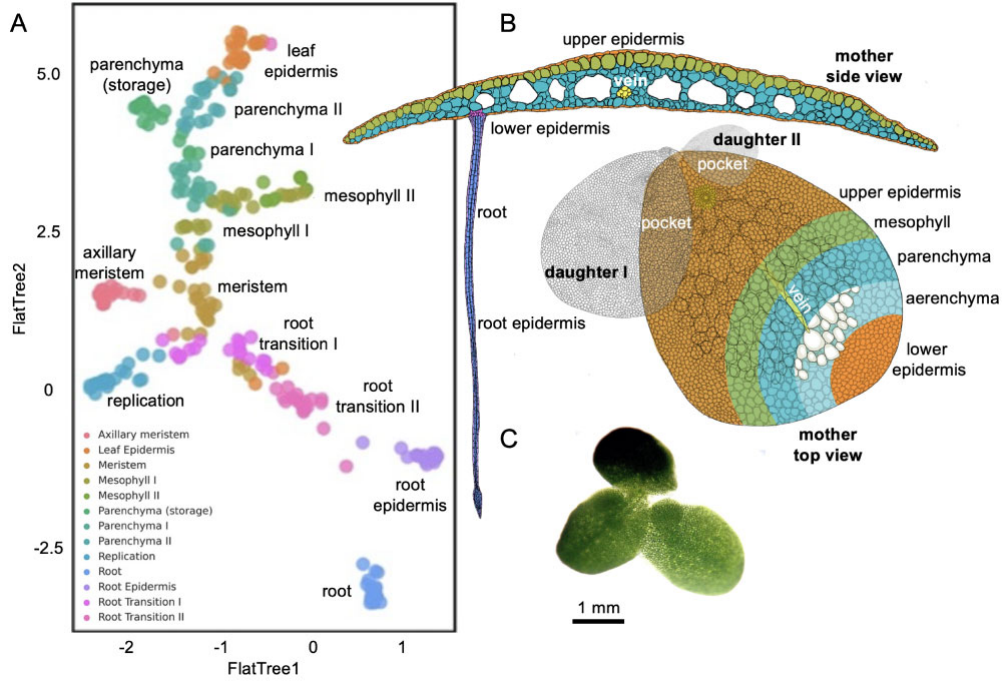
Trajectory analysis suggests 13 cell types within the *L. minuta* plant. A, UMAP tree embedding of snRNA-seq clusters describing the cell types from clusters described in Figure  5. B, Cartoon of *L. minuta* based on (Banaszek and Musiał, 2011) coloring specific cell types per the UMAP. C, Live image of lm5633 showing the connected mother and daughter fronds, with one daughter frond visible in the pocket.

GO analysis of the marker genes provided additional functional support for the distinction of each cluster as well as potential functional overlap of predicted cell types (Figure 6). Most cluster/cell types had unique GO terms while some had overlapping terms such as meristem–replication, meristem-axillary meristem, root epidermis-axillary meristem, root-axillary meristem, root transition I-axillary meristem, and meristem-root. These results suggest that the meristem-like tissue and different root cell types are intimately associated. Together with orthology from model plants (described below for each predicted cell type), our preliminary cell trajectory suggested that meristem gives rise to root transition, meristem-like, and mesophyll cells; mesophyll then gives rise to parenchyma followed by epidermis, while root transitions give rise to root epidermis cells followed by root cells (Figure 7).

### Putative meristematic-cell cluster definition

Following pseudo-time analysis, the gene expression profiles resulted in a large continuous cluster with multiple branches to terminally differentiated cell types (Figures 5, B and 7, A). In the Uniform Manifold Approximation and Projection (UMAP) embedding, we found several clusters relating to growth and division. Specifically, we defined the meristem (cluster 1) by a *TRANSLOCASE OF CHLOROPLAST 34 (TOC34)*, *ACTIN-RELATED PROTEIN (ARP4*), and the *ARGONAUT-like* marker shown to be expressed in the shoot apical meristem in maize (Xu et al., 2021). A *TOC34* promoter GUS fusion in Arabidopsis has been reported in the meristem of green tissues and root cells, and *ARP4* has been shown to function in flower development across species (Gutensohn et al., 2000; Pandey and Chaudhary, 2016). Furthermore, we surveyed the most representative GO terms for the meristem cluster and found that the top eight most representative terms relate to response to oxidative stress or removal of superoxide radicals, which could be associated with DNA damage protection (Supplemental Table S10). However, the representative set of GO terms relate to tissue development including GO:1905330, “regulation of morphogenesis of an epithelium”; GO:0090175, “regulation of establishment of planar polarity”; GO:2000023, “regulation of lateral root development”; GO:0048831, “regulation of shoot system development”; GO:0022603, “regulation of anatomical structure morphogenesis.” Finally, 11.62% of all GO descriptions related to “development,” which was the highest for all clusters, compared to terminally differentiated cell types where the lowest occurred in the putative “leaf epidermis,” containing only 3.6% of terms relating to development (Supplemental Tables S11 and S12).

The placement of the meristem cluster in the UMAP embedding and trajectory analysis was supported by two adjacent clusters described here as the “axillary meristem” and “replication” (or potentially endoreplication). The axillary meristem identification was supported by the presence of *LAX PANICLE1*, which was shown to be mediated at the transcript level in the axillary meristem in rice (Oikawa and Kyozuka, 2009). There were also several cluster marker genes involved in ribosome biosynthesis and genes coding for the ribosomal complex *(PRPL10, RLP24, RPA1A, RPL12-C*, and *RPS16)*, which would suggest these cells were actively translating mRNA into proteins. The DNA replication/endoreduplication cell cluster contained *ETHYLENE INSENSITIVE* that has been shown to control cell expansion through ethylene signaling and an *A-TYPE CYCLIN*, which is critical for G1-to-S phase transition having a central role in the meristematic tissue (Takahashi et al., 2010; Feng et al., 2015). These clusters shared common GO terms, GO:0051093, “negative regulation of developmental process”; GO:0022603, “regulation of anatomical structure morphogenesis,” which suggested they are cells with defined processes similar to meristematic cells but different enough to warrant exclusive clustering.

The meristem cells give rise to all other cell types in clonally propagated plants, yet many of the transcription factors and networks leading to fated cell types are unknown. Transcription factor *WRKY32* had increased expression in the transition from meristem to green frond-like cell types, where it has been previously shown to be involved in ethylene signaling in tomato (*Solanum lycopersicum)*, where RNAi repression leads to yellowing of fruits (Zhao et al., 2021). It has been shown previously that related *L. minor* clones show reduced growth from exogenous ethylene treatment (Utami et al., 2018). Conversely, WRKY transcription factors associated with the root cell transition are *WRKY6* and *WRKY65*. *WRKY6* is associated with pathogen defense, phosphate translocation, and arsenate resistance and *WRKY65* induces Jasmonate and salicylic acid responses relating to pathogen response (Huang et al., 2016; Wang et al., 2020). The expression of several WRKY transcription factors at the meristematic transition leading to terminal cell types suggested hormone signaling was a major contributing factor in lm5633 cellular development. Further, in vivo characterization will be required to determine the involvement of these transcription factors in cellular development.

### Putative root-like tissue cluster definition

The predicted meristem-like cells were centrally located between two branches leading to terminal cell types in the trajectory analysis (Figures 5, B and 7, A). Although it has not been confirmed that Duckweed roots are essential or act similarly to well-developed root systems in model plants, here we hypothesized root cell types by maker genes involved in metabolite transport by *HIGH-AFFINITY POTASSIUM TRANSPORTER 8* and *MANGANESE ATPASE TRANSPORTER* *(*Mills et al., 2008). We found an additional marker gene *UBIQUITIN E2 CONJUGATING ENZYME* that has been shown to be involved in root development (Wen et al., 2014). Finally, two copies of the tandemly duplicated *PLANT LIPOXYGENASE 9* (*LOX9*) gene were found in root-like cell types, where *LOX9* has been shown in soybean to be expressed in root nodules (Hayashi et al., 2008). We also found an additional paralog of *LOX9* in the root transition I cluster suggesting the paralogs may be cell specific but concordant in their function progressing toward formation of root-like cells. These marker genes provided specific functional roles associated with root cell types yet require in vivo validation of expression patterns and a careful biochemical analysis to validate the annotated functional role.

Visual morphologies have historically been used to define long-lived, stable cell types. Single-cell/nuclei sequencing aims to capture all cell types including transitory cells that may not appear visually distinct yet are molecularly distinct. In some cases, snRNA-seq may capture transitional cell types that do not have well characterized visual properties or associated marker gene expression profiles but distinct transitional cell types may show progression to terminal cell types based on mRNA profiles. Although difficult to define a specific cell type, here, the root transition II cluster contained a marker gene for *ASYMMETRIC LEAVES1*, which is essential for adaxial–abaxial leaf polarity and associated with an initial committed step toward root-like tissue development (Xu et al., 2003; Machida et al., 2015). Likewise, *SLEEPY1* is responsible for gibberellin signaling, cell growth, and elongation, suggesting this cluster contained cells transitioning to the final root-like cells (McGinnis et al., 2003; Xu et al., 2003)*. ELONGATED HYPOCOTYL 5* was also found in this cluster and has been shown to play a role in induction of light-induced genes and root gravitropism (Srivastava et al., 2015). It is of note that lm5633 roots were exposed to light in this experiment since the plants are grown in clear flasks, which resulted in the roots appearing visually green and expressing light-harvesting genes (Figure 7C).

### Putative epidermal tissue cluster definition

The exterior cells forming the epidermis provide a barrier from the rhizosphere and phyllosphere, and are often associated with increased production of hydrophobic waxes and cutins. Lipid biosynthesis is essential for wax and cutin production that provide extracellular protection in epidermal cells. Fatty acid biosynthesis, known to occur in epidermal cells is mediated through the key marker genes *3-KETOACYL-COENZYME* and *LONG CHAIN ACYL-CoA SYNTHETASE* (Kim et al., 2013). Additionally, root epidermal cells were defined based on expression of *CRINKLY 4*, which is known to have a role in maize epidermal cell formation (Becraft et al., 1996). Frond epidermal cells were located in close proximity to root epidermal cells in UMAP embedding consistent with these cell types sharing similar expression despite having different cell trajectories (Figures 5, B and 7). The frond epidermal cells were defined by the marker gene *ECERIFERUM-like*, which has been shown to be important for cuticle wax development (Haslam et al., 2017). Additional markers included an auxin efflux carrier in starch metabolizing cells *PIN-FORMED (PIN7)* aiding root termination (Kim et al., 2013; Rosquete and Kleine-Vehn, 2018). This was consistent with this cluster’s position in the UMAP embedding (Figure 5B) in close proximity to the root epidermis but highly diverged in the complement of expressed photosynthetic genes and complete divergence in the trajectory analysis (Figure 7A). Likewise, this cell type contained genes *ATP-DEPENDENT ZINC METALLOPROTEASE* (*FTSH2)*, two orthologs of *DEFECTIVE KERNEL 1 (DEK1)*, and *CYTOCHROME C OXIDASE 15* (*COX15)*. These genes are involved in carbon storage where *FTSH2* has been shown to be involved in thylakoid biosynthesis and *COX15* shows increased expression in response to decreased cellular respiration (Vishwakarma et al., 2015; Haslam et al., 2017). Similarly, *DEK1* is expressed in aleurone-like cells in maize that are involved in starch metabolism (Tian et al., 2007). It has been shown that *DEK1* also has adverse effects on leaf morphology and it is likely that this cluster contained frond epidermal cells. Finally, we combined the root and frond epidermis marker gene GO terms and determined that the six most representative are: GO:0048580, “regulation of post-embryonic development”; GO:0044249, “cellular biosynthetic process”; GO:0009117, “nucleotide metabolic process”; GO:0006753, “nucleoside phosphate metabolic process”; GO:0019219, “regulation of nucleobase-containing compound metabolic process”; GO:2000112, “regulation of cellular macromolecule biosynthetic process.”

### Putative frond tissue cluster definition

Mesophylls, located in leaf or frond tissue, are the primary cells involved in photosynthetic light and CO_2_ capture for the generation of sugars to sustain the plant. Mesophyll-like cells were determined based on substantial expression of multiple marker genes associated with light perception, thylakoid biogenesis, and cytokinin degradation related to stress by *YELLOW STRIPE-LIKE (YSL9)* and *THYLAKOID LUMEN PPIASE* (*TLP40*). *YSL9* in rice is associated with leaf vasculature but in this case is potentially more similar to mesophyll-like cells in this basal aquatic monocot (Wen et al., 2020). *TLP40* has been shown to be expressed in mesophyll and at two-fold greater expression in bundle sheath cells (Vojta et al., 2019). The representative GO terms suggested these cells are carbon limited, and the photorespiratory cycle is engaged where the top GO terms are: mitochondrial transport of glycolate (GO:0006626, “protein targeting to mitochondrion”; GO:0072655, “establishment of protein localization to mitochondrion”; GO:1901975, “glycerate transmembrane transport”; GO:0097339, “glycolate transmembrane transport”).

Parenchyma cells in any given tissue can become specialized for a variety of purposes, yet in lm5633 it appeared the specialization of parenchyma was geared toward photosynthetic metabolism and storage of photosynthate. Parenchyma I and II clusters were defined by *EXORDIUM* *(EXO)-like*, *SUCROSE SYNTHETASE 1 (SUS1)*, *STARCH EXCESS 4 (SEX4)*, and *XAP5 CIRCADIAN TIMEKEEPER*, which promotes ethylene response in aerial tissue (Ellison et al., 2011). *SUS1* is a prominent player in the accumulation of terminally synthesized photosynthate in source tissues and *SEX4* mutants in leaf tissues promote starch metabolism (Kötting et al., 2009; Ma et al., 2014; Shi et al., 2019). Mutants of *EXO* have decreased epidermis, palisade, and spongy parenchyma in Arabidopsis, which suggests *EXO* may be inhibiting differentiation to a terminal cell type (Florian et al., 2009; Schröder et al., 2011). Marker genes in these clusters pointed to photosynthetic metabolism. A terminal cluster was observed branching off the parenchyma-like cells (Figure 7A) denoted here as “parenchyma (storage)” since cells are expressing two copies of *ALPHA AMYLASE 2* and *IMBIBITION INDUCIBLE.* The combination of these marker genes in a cell type may mean these cells were actively metabolizing starch in the vacuoles.

### Specific cell expression associated with invasiveness from preliminary snRNA-seq

**Figure 8:**
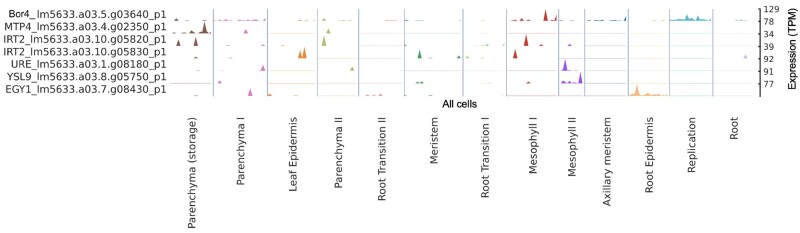
Metal transport and accumulation-associated genes in *L. minuta* are expressed in putative mesophyll cells. snRNA-seq expression profiling of metal tolerance-related genes suggest a cell-type-specific expression pattern with greater gene expression localized to green tissue types in lm5633. Each peak represents transcript expression of the respective gene within each predicted cell type. The scale (right; *y*-axis) shows the range of gene expression (TPM), and all of the cells are on the *x*-axis. For example, Lm5633.a03.5.g03640 (top) encodes *BOR4* localized to mesophyll and replicating cells described further in the text.

*Lemna minuta* can occupy a variety of ecosystems due to its adaptability to absorb and tolerate an excess of micro- and macro-nutrients. The uptake and adaptability of *L. minuta* has led to its use as a wastewater detoxifying/phytoremediation species (Fernández et al., 2020). Wastewaters are often contaminated with a variety of toxic compounds yet the exact mechanisms of plants ability to adapt to these compounds, including high levels of essential elements and heavy metals, remains unclear (Frick, 1985; Davis et al., 2002; Del-Campo Marín and Oron, 2007; Gür et al., 2016; Liu et al., 2018; Türker et al., 2019). We found increased expression of *BORON TRANSPORTER 4* (*BOR4*) in the mesophyll and replicating cells (Figure 8). We also found a large TD (11 copies; eighth largest) of a boric acid transporter (MIP aquaporin) (Figure 3C; Supplemental Table S4). We found similar increased expression of *IRON-REGULATED TRANSPORTER 2* (*IRT2*) and *METAL-NICOTIANAMINE TRANSPORTER 9* in the mesophyll cells. *IRT2* also showed a TD expansion (four copies with two copies expressed here), although modest compared to *BOR4*. Overaccumulation of boron and toxic heavy metals can have an adverse effect on plant growth yet *L. minuta* grows and accumulates heavy metals readily (Gür et al., 2016). This was compelling evidence that *L. minuta*’s invasiveness and adaptability in wastewater may be, at least in part, due to the increased *BOR4* expression, as *BOR4* is essential for boron export from cells. Additional snRNA-seq under micro- and macro-nutrient stress conditions would greatly improve our understanding of cell-specific responses and adaptation to abiotic stressors.

## Discussion

Here we endeavored to define the trajectory of cells in a morphologically reduced, fast-growing nonmodel plant useful for phytoremediation but also invasive in specific environments. We generated a chromosome-resolved genome for the Lesser Duckweed (*L.* *minuta*) clone lm5633 and found that it did not share a lineage-specific WGD with *S. polyrhiza* yet had an increased number of TDs for genes involved in pathogen defense, response to stress and nutrient acquisition. Additionally, we performed low cell coverage SMARTseq2-based snRNA-seq on a clonal population of whole lm5633 plants representing all developmental stages, which enabled us to hypothesize cell types based on several computational criteria as well as the supposed cellular developmental trajectory from the meristematic cells. While this dataset was based on a limited number of nuclei, it provided expression support that the cells we are computationally defining as mesophyll may be a site of elemental and heavy metal transporter expression important for phytoremediation and invasiveness.

Duckweed genomes provide a compelling platform for gene annotation and functional analysis. It was shown that *S. polyrhiza* has a reduced set of nonredundant protein-coding genes even though it has had two lineage-specific WGD events (Wang et al., 2014). One challenge in model species and crops is the number of paralogs (family size) that complicate gene functional analysis due to redundancy of action. Here, the lm5633 genome also has a streamlined set of genes that shows a similar reduction in paralogs compared to other plants (Figure 4A). We find evidence that both lm5633 and wa8730 lack the *S. polyrhiza* lineage-specific WGD and only have the shared monocot τ WGD (Figure 4, B and C). Currently there are conflicting reports whether *S. polyrhiza* has the τ WGD (Wang et al., 2014; Ming et al., 2015; Hasing et al., 2020). One explanation is that the lineage-specific WGD has obscured the τ WGD in *S. polyrhiza*, and that lm5633 is more fractionated due to the very ancient (∼150 mya) WGD. More Duckweed genomes will help resolve the WGD history.

Duckweed are particularly suited for single-cell studies due to their size, direct contact with the media, small nonredundant genome, and rapid clonal growth (Figure 1). Most single-cell studies to date have been conducted in model plants using protoplasting on root or reproductive structures, which have the advantage of having all cells in a specific developmental continuum (Denyer et al., 2019; Ryu et al., 2019; Liu et al., 2020a). Similarly, Duckweed provides a continuum of all cell types and developmental stages due to their rapid clonal growth and nested generations and minimal body size (Figure 1). Moreover, Duckweed is in direct contact with their media allowing for detailed and controlled manipulation of the environment, which in the past was leveraged to understand auxin biology (Slovin and Cohen, 1988).

Since our goals in this study were to identify genes associated with phytoremediation and invasiveness in a nonmodel system, we made specific experimental design decisions that resulted in trade-offs that impact interpretation of the results. First, the snRNA-seq method described here couples well with experiments where abiotic/biotic treatments of the samples are desired because they can be immediately frozen and transcriptomic perturbations measured; additionally frozen nuclei presumably capture actively transcribing mRNA. One potential downside of snRNA-seq is the loss of RNA in the cytoplasm; more experiments comparing the different techniques will be required to evaluate this limitation. Second, in this study, we used the SMARTseq2 to identify a higher number of transcripts per nuclei, but due to the manual nature of this approach we sampled many fewer nuclei (100s of cells) compared to other methods like 10X genomics (1,000s of cells). A limited number of sequenced nuclei in this study may not adequately survey the continuum of all possible unique terminally differentiated cell types and transition cell types. Certainly, greater depth in the number of unique cells sequenced will provide insight into the absolute number of terminal and transition cell types. For example, in this snRNA-seq study, it is unclear if the numbers of cells detected for each cell type accurately reflect the numbers of cells expected in whole plants in vivo but could be determined with a larger number of replicate sequencing runs.

All single-cell studies are currently in model systems, yet as we have shown here one can go from a wild collected plant to a chromosome-resolved genome and leverage snRNA-seq dataset to generate functional predictions. While model plants and specifically Arabidopsis have high quality annotations and marker genes for cells, there are substantial challenges in defining marker genes or assigning cellular functions for even well studied plants and crops. Here we leveraged a multi-tiered approach to assign cell types from snRNA-seq clusters. Our realization was that cell trajectory analysis provides rich information about cell type, especially when it is coupled with a developmental series using an entire Duckweed plant as shown in this study or has been shown in model plants with discrete tissues, like roots and floral tissues (Denyer et al., 2019; Satterlee et al., 2020; Xu et al., 2021). GO and orthology comparisons are fraught with issues due to sparse datasets (i.e. few or no GO terms associated with gene predictions) yet coupled with predicted maker genes the GO terms can help in determining cluster uniqueness and potential functional role of predicted cell types (Figure 6) and this is useful when orthology to model organism is not present. However, when multiple criteria are leveraged, annotation can be improved as well as provide support for known and potentially new cell types, like transition cell types. While combined computational approaches are powerful to define cell types, the cell types and trajectories described here are a good starting point but will require further in vivo molecular characterization.

As cell types have generally been defined based on the cell’s physical morphology, single-cell/nuclei RNA sequencing studies offer a molecular-based approach to define transitioning cell types. The definition of cell types presents a problem when trying to describe the snRNA-seq molecular dataset as sub-populations of cell types that may exist within one visibly distinguishable cell population. In this respect, the expression data become the molecular definition of a cell, cell transition, or cell fate. One might expect that for a clonally propagating plant with nested daughter and GD plants maturing internally that the number of cells with different global transcriptomic profiles could be continuous, or that cell types that defy previous visual definition or detection will be encountered. Therefore, assigning known cell types to expression signatures may be an artificial step to facilitate general understanding. Future studies when more single-cell datasets are available in both nonmodel and model systems may define cell types solely on expression profiles.

The trajectory of cell types from meristem to terminally differentiated cells may provide clues as to their function. Cellular studies have suggested that the meristem is unique in Duckweed since it gives rise to daughter fronds (Landolt, 1986). We found the putative meristem cell type is divided into three potential cell clusters (meristem, replication, and axillary meristem), which provides a clue as to how Duckweed is dividing rapidly leading to continuous daughter frond production. The overlapping trajectory and GO terms of mersitem- and root-like cells (Figures 6 and 7), suggest the root may play additional roles that are unknown at this time, and may clarify its usefulness. Furthermore, root and frond epidermal cells have distinct trajectories in this preliminary study (Figure 7) yet arrive in a similar expression space consistent with their overall cellular function (Figure 5B). Finally, photosynthetic mesophyll-like cells emerge from the meristem then give rise to the metabolically active and terminally differentiated parenchyma cells. We did not identify stomatal cells or vascular tissue (vein) cells and this could be a result of our methodology which resulted in a limited cell population captured, or the fact that *L. minuta* only has one vein and about 30 stomata (∼60 cells per plant) (Landolt, 1986). Since each mother frond has 256 (each daughter frond has two or more generations) developing, yet unexpanded daughter/GD fronds, the majority of the cells we detect will be actively dividing; deeper sequencing will be required to accurately identify small populations of terminally differentiated cells.

In model plants, nutrient uptake and transport primarily occur in the roots and vasculature (Yan et al., 2020). However, the Duckweed frond is in direct contact with its environment, which may explain why, in this initial snRNA-seq dataset, we detect high expression of nutrient uptake genes in the cells we define as the photosynthetically active mesophyll (Figure 8). Both *ITR2* and *BOR4*, which are also both expanded in lm5633 through TDs, are highly expressed in mesophyll. However, it has been shown that *IRT2* is expressed in the roots (Vert et al., 2001), and *BOR4* is expressed in the xylem in Arabidopsis (Takano et al., 2002). The solitary vein of lm5633 is in close proximity to the mesophyll, which makes it formally possible that we have mis-identified some of these cells and they actually are vasculature or at an expression level too similar to separate with the current sequencing coverage and sequenced cell population. Either way, this simplified system provides an opportunity to further define the acquisition, transport, and storage of toxic compounds at a single-cell level. Future studies will enable clarification of cell types as well as provide the opportunity to dissect the specific cellular mechanisms associated with phytoremediation and invasiveness.

## Conclusions

Duckweed provide an unprecedented opportunity to study the cell fates across an entire morphologically reduced plant. Coupled to snRNA-seq, this system provides an opportunity to study the cell-specific responses to environmental changes. This dataset will surely be refined with additional datasets in additional plants. Different plants, conditions, and treatments will refine our understanding of molecular cell types and ultimately identify additional cell types, transition states, and niches.

## Materials and methods

### Plant collection and growth

The Lesser Duckweed (*L.* *minuta*) was collected on October 10, 2019 from Cotton Creek Park, Encinitas, CA, USA (33°2′58″N 117°17′29″W), which is a waste water slough close to the ocean (Figure 1A). A population of clones were collected, surface sterilized and one clone was retained to multiply the population. The representative clone was deposited in the RDSC (www.ruduckweed.org) and was assigned the clone number lm5633. Plants were propagated on Schenk and Hildebrandt media as described at RDSC.

### Genome sequencing

High molecular weight (HMW) DNA was extracted with modifications (Lutz et al., 2011). The resulting HMW DNA was quality controlled for size and contamination. Unsheared HMW DNA was used to make ONT ligation-based libraries. Libraries were prepared starting with 1.5 µg of HMW DNA and following all other steps in ONT’s SQK-LSK109 protocol. Final libraries were loaded on a ONT flow cell (version 9.4.1) and run on the GridION. Bases were called in real-time on the GridION using the flip-flop version of Guppy (version 3.1). The resulting fastq files were concatenated and used for downstream genome assembly steps. A total of 3,555,475 reads for a total length of 9,874,060,222 bp resulted in approximately 27× coverage for the ONT data. Illumina 2×150 paired-end reads were also generated for genome size estimates and polishing genome sequences. Libraries were prepared from HMW DNA using NEBNext Ultra II (NEB, Beverly, MA, USA) and sequenced on the Illumina NovaSeq. Resulting raw sequence was only trimmed for adaptors, resulting in 183,906,622 reads with a total sequence length of 55,245,330,271 bp for a total of 153 × coverage.

### Genome size estimation

K-mer (*k* = 31) frequency was estimated with Illumina paired-end reads (2 × 150 bp) using Jellyfish (version 2.3.0; Marçais and Kingsford, 2011) and analyzed with GenomeScope and GenomeScope2 (Vurture et al., 2017; Ranallo-Benavidez et al., 2020). Genome size, heterozygosity, and repeat content were first estimated with GenomeScope (http://qb.cshl.edu/genomescope; Table 1). The K-mer frequency plot is consistent with a highly heterozygous diploid genome or a tetraploid genome; based on previous flow cytometry, *L. minuta* is diploid with an average genome size of 365 Mb (Table 1; Bog et al., 2020).

### Genome assembly

Resulting fastq files passing  quality control (QC) were assembled using our previously described pipeline (Michael et al., 2018) with the modification that the initial assembly was generated using FlyE (Kolmogorov et al., 2019). The resulting assembly graph was visually inspected with Bandage (version 0.8.1) (Wick et al., 2015), which revealed a branching pattern consistent with a heterozygous genome with structural differences between haplotypes. A consensus sequence was generated with three iterative cycles of mapping the ONT reads back to the assembly with minimap2 followed by Racon (version 1.3.1; Vaser et al., 2017), and the final assembly was polished iteratively 3 times using 2 × 150-bp paired-end Illumina reads mapped using minimap2 (version 2.17-r941) (>98% mapping) followed by pilon (version 1.22; Walker et al., 2014). The resulting assembly was assessed for traditional genome statistics including assessing genome completeness with Benchmarking Universal Single-Copy Orthologs (BUSCO) version 3 liliopsida odb10 database (Table 1; Simão et al., 2015). In order to address the uncollapsed heterozygosity in our draft assembly, evident from both high duplication of *BUSCO* genes and inspection via bandage, we processed the assembly using Purge Haplotigs in order to generate a haploid assembly (Roach et al., 2018). The amount of purged heterozygosity was estimated as described by KAT (Mapleson et al., 2017; Supplemental Figure S2).

### High-throughput Chromatin Conformation Capture (HiC) genome scaffolding

Crosslinking was performed on ground tissue and nuclei were isolated following (Colt, 2021). HiC data were generated using the Arima-HiC Kit User Guide for Plant Tissues (Link et al., 2018), according to the manufacturer’s protocols. Libraries were generated following the Arima-HiC Kit Library Preparation Guide for Swift Biosciences Accel-NGS 2S Plus DNA Library Kit and sequenced on Illumina NovaSeq. A total of 208,822,590 reads with a total sequence length of 62,095,632,959 bp were collected for a total of 172 × coverage. We used standard methods defined in the Aiden lab genome assembly cookbook (https://github.com/aidenlab/3d-dna/). The primary steps include alignment of HiC reads to the haplotype purged lm5633 draft assembly and creation of a contact map using the Juicer pipeline followed by automated scaffolding using 3D-DNA (Durand et al., 2016b). The scaffolds were then inspected and manually corrected with Juicebox Assembly Tools before being finalized by the 3d-dna postreview pipeline (Durand et al., 2016a; Dudchenko et al., 2017). The resultant scaffolds for lm5633 were output and renamed from longest to shortest.

### High copy repeat analysis

Long read ONT assemblies provide another measure of completeness through the identification of high copy repeats such as centromeres and telomeres sequences (VanBuren et al., 2015). We employed a searching strategy to identify the centromeres that leverages the idea that the highest copy number tandem repeat (TR) will be the centromere in most genomes (Melters et al., 2013). We searched the genomes using TR finder (version 4.09) using modified settings (1 1 2 80 5 200 2000 -d -h) (Benson, 1999). TRs were reformatted, summed, and plotted to find the highest copy number TR per our previous methods (VanBuren et al., 2015). While lm5633 had robust telomere arrays (Table 1), we could not detect a high copy number centromere repeat similar to what we have found in *S. polyrhiza* (Michael et al., 2017), which could mean lm5633 has holocentric centromeres.

### Gene prediction and annotation

The chromosome resolved lm5633 genome was annotated using a pipeline consisting of four major steps: repeat masking, transcript assembly, gene model prediction, and functional annotation. Repeats were identified using ethylenediamine tetraacetic acid  (EDTA) (version 1.9.8; Ou et al., 2019) and these repeats were used for softmasking. ONT cDNA reads were aligned to the genomes using minimap2 and assembled into transcript models using Stringtie (version 1.3.6). The softmasked genome and Stringtie models were then processed by Funannotate (version 1.6; https://github.com/nextgenusfs/funannotate) to produce gene models. The resulting gene models were renamed reflecting the chromosome and the linear position on the chromosome. Predicted proteins were then functionally annotated using Eggnog-mapper (version 2; Huerta-Cepas et al., 2017).

### Orthogroup analysis and synteny

Gene families and overrepresented groups were determined with Orthofinder (version 2.4.0) and CAFE5 (https://github.com/hahnlab/CAFE5). Genomes were accessed from Phytozome13 (https://phytozome-next.jgi.doe.gov/) or from specific publications such as the *Colocasia esculenta* (Taro) (Yin et al., 2021), *S.* *intermedia* (Hoang et al., 2020), *S.* *polyrhiza clone 9509* (Hoang et al., 2018), and *W.* *australiana* clone 8730 (Michael et al., 2020). Orthofinder results were parsed and used for CAFE5 by modifying the species tree with “make_ultrametric.py” and filtering orthocounts.tsv with “clade_and_size_filter.py.” Gene trees were visualized with iTOL (Letunic and Bork, 2019). The SynMap tool on CoGe (Grover et al., 2017) and McScan python version (https://github.com/tanghaibao/jcvi/wiki/MCscan) were utilized to generate whole genome synteny maps, identify syntenic orthologs, estimate synonymous substitution rate (Ks) across genomes, and generate figures.

### snRNA-seq

We generated an snRNA-seq dataset from a population of whole sterile lm5633 plants grown in 250 mL erlenmeyer flasks under 12 h of light and 12 h of dark (intermediate days) with constant 22°C temperature (light:dark:hot:hot)*.* SnRNA-seq was performed using the SMARTseq2 protocol on nuclei isolated from frozen plant material as previously described (Bakken et al., 2018) with some modifications. Nuclei were extracted with a custom nuclei extraction buffer consisting of Tris–Hcl (pH 9.5) 15 mM, EDTA 10 mM, KCl 130 mM, NaCl 20 mM, PVP-10 8%w/v, Spermine 0.07 g, Spermidine 0.05 g, Triton X-100 0.10%v/v, BME 7.50%v/v. Individual nuclei were sorted on the FACsAria Fusion system into SMARTseq2 lysis buffers allocated in 3 separate 96-well plates. The resulting libraries were sequenced on the Illumina NovaSeq platform collecting 116,435,500,000 bp equating to 6,672× coverage of the transcriptome and an average of 23× coverage per nuclei.

Reads were mapped to the genome and quantified using Salmon to produce an expression matrix (Supplemental Table S13). We leveraged Single-cell Trajectories Reconstruction, Exploration, And Mapping (STREAM) to perform quality filtering, normalization, dimensionality reduction, visualization, clustering, differential expression, and trajectory analysis (Chen et al., 2019). Lowly expressed genes and genes expressed in fewer than five cells, were removed from the analysis. Low quality cells, cells with fewer than 20,000 reads mapped or fewer than 100 genes expressed were filtered out; this quality filtering resulted in 269 cells with 8,457 genes expressed. Expression data was filtered using st.filter_cells(adata,min_n_features = 100), st.filter_features(adata,min_n_cells = 5), and st.filter_cells(adata,min_n_counts = 20,000) and normalized as st.normalize(adata,method = ‘lib_size’) and st.log_transform(adata). Initial QC was calculated as st.cal_qc(adata,assay = ‘rna’) and scanpy.pp.calculate_qc_metrics(adata, percent_top = None, log1p = False, inplace = True). Variable genes were determined but ultimately all genes were used for principal component analysis and clustering. The top 100 principal components were used with code st.select_top_principal_components(adata,feature = ‘var_genes’,first_pc = True,n_pc = 100). Clustering was performed with K-means clustering. We searched all values of *K* = 2 to 100 and chose *K* = 13 as it maximized our silhouette score while maintaining the largest number of cells per cluster. Final clustering was performed as st.dimension_reduction(adata,method = ‘umap’,feature = ‘top_pcs’,n_components = 2,n_neighbors= 26,n_jobs = 4) and st.seed_elastic_principal_graph(adata,n_clusters = 13) and trajectory analysis as st.elastic_principal_graph(adata,epg_alpha = 0.01,epg_mu = 0.05,epg_lambda = 0.05,jobs = 10) and st.extend_elastic_principal_graph(adata, epg_ext_mode = ‘WeigthedCentroid’,epg_ext_par = 0.8). Marker genes were then determined as st.detect_markers(adata,ident = ‘kmeans’,marker_list = adata.uns['var_genes'],cutoff_zscore = 1.0,cutoff_pvalue = 0.01,n_jobs = 10,use_precomputed = False) and transition markers as st.detect_transition_markers(adata,marker_list = adata.uns['var_genes'],cutoff_spearman = 0.4,cutoff_logfc = 0.25,n_jobs = 10,use_precomputed = False). SCANPY was used for data visualization purposes (Wolf et al., 2018). Plotting tools and methods are described in detail by STREAM and SCANPY methods.

The 269 nuclei formed 13 clusters that we defined using GO, KEGG, and PFAM annotation into cell types based on orthologous marker genes. Orthology was determined with Orthofinder (Emms and Kelly, 2019) as above. Marker genes from model species based on existing literature were used to search the orthogroups for lm5633 orthologs (Supplemental Table S8). The marker genes in model organisms were compared with the orthofinder gene table and a corresponding lm5633 ortholog was assigned. Generally, annotations with a geneID from eggnog mapper were more reliable than observing marker genes in larger gene families (>3 copies per genomes). GO terms and quantities for cluster marker genes were parsed to form marker genes lists. GO lists were visualized using REVIGO and CirGO (Supek et al., 2011; Kuznetsova et al., 2019).

### Accession numbers

The final genome assembly is available on CoGe (https://genomevolution.org/) under the ID:61245 or Biosample: SAMN19243672. Genomic and snRNA-seq reads can be found in SRA under Biosample: SAMN19243672.

## Supplemental data 

The following materials are available in the online version of this article.

**Supplemental Table S1.** BUSCO scores for lm5633.

**Supplemental Table S2.** Predicted repeat types in the lm5633, sp9509, and wa8730 genomes.

**Supplemental Table S3.** A comparison of telomere sequence lengths in Duckweed.

**Supplemental Table S4.** TDs in lm5633.

**Supplemental Table S5.** Read mapping statistics per cell.

**Supplemental Table S6.** Read mapping statistics per gene.

**Supplemental Table S7**. Previously published marker genes used for defining snRNA-seq cell types.

**Supplemental Table S8.** Orthogroups across all species.

**Supplemental Table S9.** *Lemna minuta* (lm5633) snRNA-seq marker genes per cell type.

**Supplemental Table S10.** GO terms for the meristem cluster.

**Supplemental Table S11.** Percentage of cluster GO terms relating to development.

**Supplemental Table S12.** GO terms associated with cluster IDs.

**Supplemental Table S13.** Raw expression matrix of cells by genes.

**Supplemental Figure S1.** *Lemna minuta* raw HiC contact map before Purge Haplotigs.

**Supplemental Figure S2**. KAT plots describing heterozygosity before and after Purge Haplotigs.

**Supplemental Figure S3.** *Lemna minuta* (lm5633) has an increased number of paralogs per orthogroup.

**Supplemental Figure S4.** Syntenic depth between lm5633, sp9509, and the basal plant Amborella.

**Supplemental Figure S5.** *The S. intermedia genome (si7747) is syntenic with lm5633.*

**Supplemental Figure S6.** Some regions of the sp9509 genome retain a 4:1 syntenic ratio with lm5633.

**Supplemental Figure S7.** Quality control of raw and post quality filtering of the expression matrix.

**Supplemental Figure S8.** Quality control of gene variance and PCA analysis.

**Supplemental Figure S9.** Cell expression profiles of marker genes for identifying cell types.

**Supplemental Figure S10.** The most significant marker genes for each cluster are highly specific.

## Supplementary Material

kiab564_Supplementary_Data

## Notes

T.P.M. and B.W.A. conceived the study. B.W.A. and M.N. carried out the lab work. K.C. performed sequencing and B.W.A., B.D.A., N.T.H., and T.P.M. performed the analysis. R.H.S. and T.P.M. oversaw the project, and T.P.M. and B.W.A. wrote the manuscript.

The author responsible for distribution of materials integral to the findings presented in this article in accordance with the policy described in the Instructions for Authors (https://academic.oup.com/plphys/pages/general-instructions) is: Todd P. Michael (toddpmichael@gmail.com).
