# Supplementary material for: The genome and preliminary single-nuclei transcriptome of Lemna minuta reveals mechanisms of invasiveness
Source: Plant Physiol. 2021 Dec 6;188(2):879–97. doi: 10.1093/plphys/kiab564 (PMC8825320; doi:10.1093/plphys/kiab564)
Supplement: kiab564_Supplementary_Data [file kiab564_supplementary_data.zip › Lemna_minuta_genome_scRNA_SuppFigures_final.pdf]

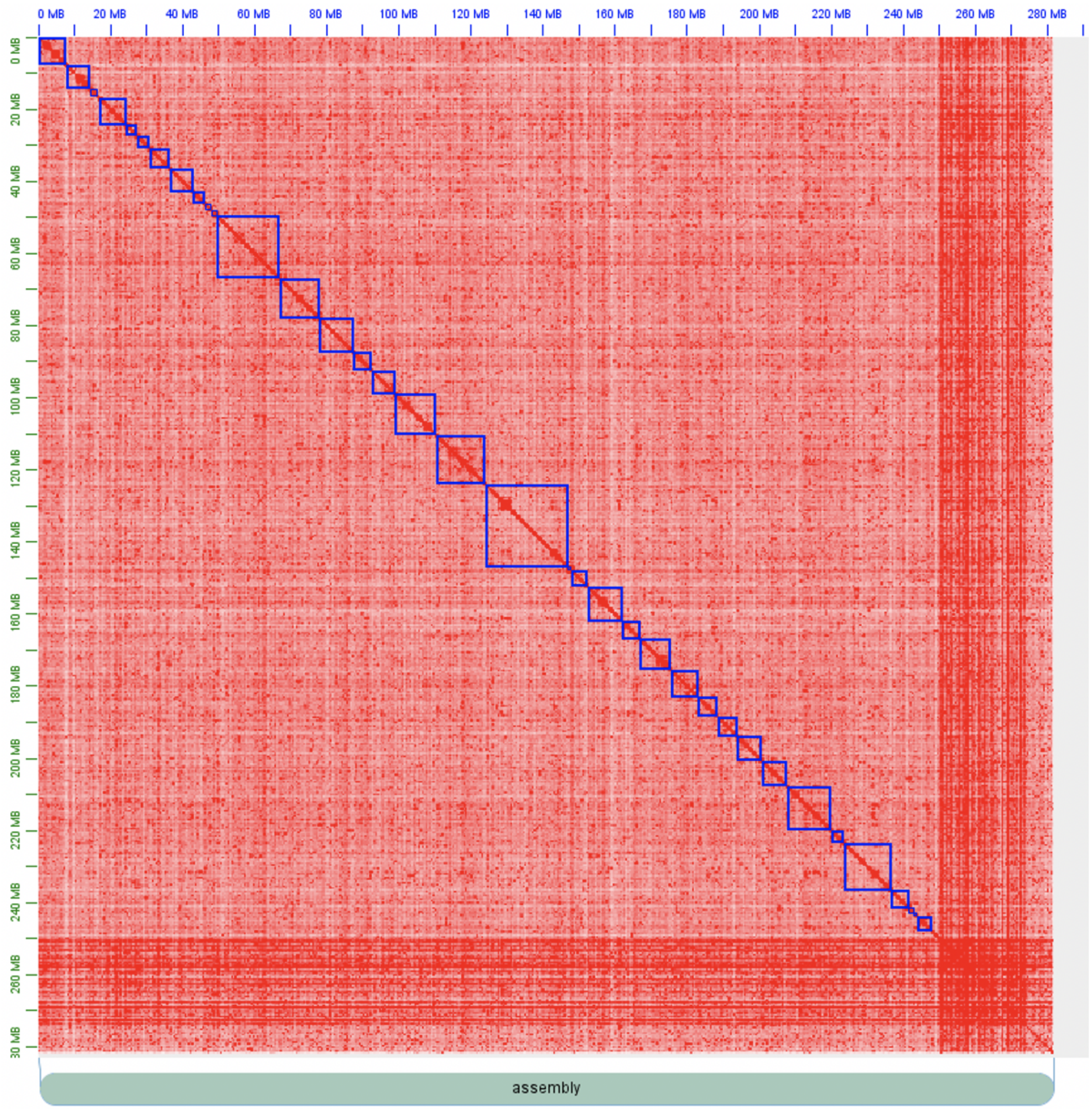

Supplemental Figure S1. ***Lemna minuta*** **raw HiC contact map before Purge Haplotigs.** High throughput chromatin conformation capture (HiC) contact map of the draft lm5633 genome assembly before removing residual haplotypes using Purge Haplotigs. Blue boxes denote potential chromosomes, while darker red represents more contacts, and lighter red to white less contacts. The dark red region, not in boxes in the bottom right, represents repeats and residual haplotypes. No manual correction was performed.

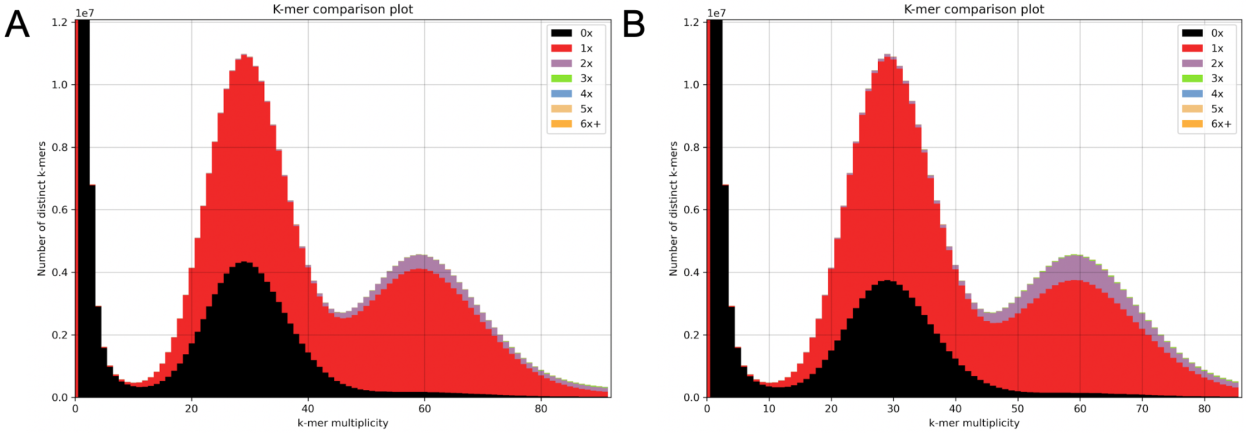

Supplemental Figure S2. KAT plots describing heterozygosity before and after Purge Haplotigs. (A) Prior to running Purge Haplotigs the 0x content peak within the first k-mer peak was about 4.2 M, while (B) after running purge haplotigs the 0x content peak within the first k-mer peak was reduced to 3.85 M consistent with the removal of the residual heterozygosity in the Im5633 genome assembly.

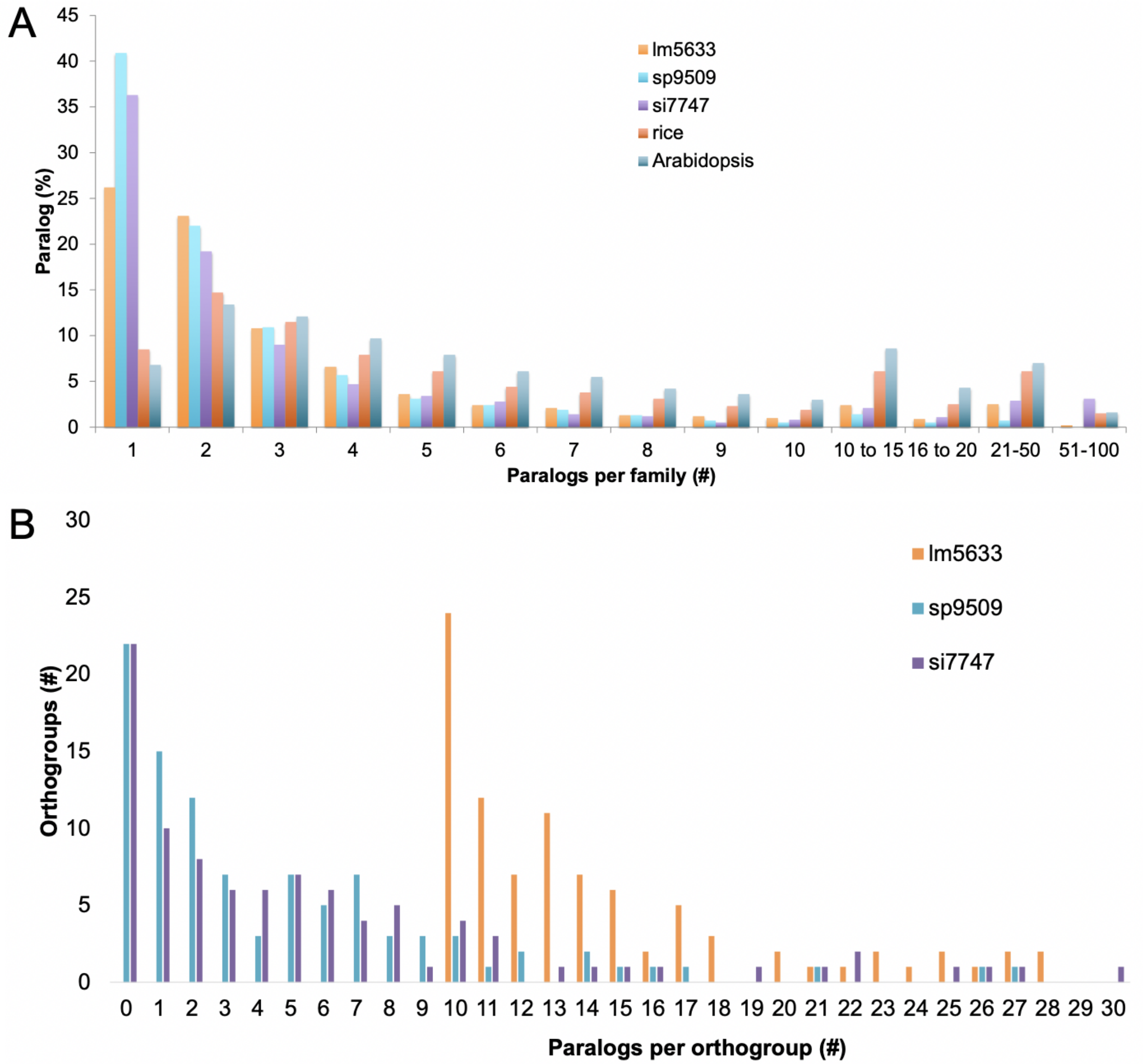

Supplemental Figure S3. *Lemna minuta* (lm5633) has an increased number of paralogs per orthogroup. A) Rice and *Arabidopsis* have fewer genes with one paralog per orthogroups compared to lm5633, sp9509 and si7747; lm5633 has a smaller percentage than sp9509 and si7747. As a result, lm5633 has more orthogroups with multiple genes per orthogroup like rice and Arabidopsis. B) Orthogroups where lm5633 has more than 10 paralogs compared to sp9509 and si7747, revealing the gene expansions are specific to lm5633.

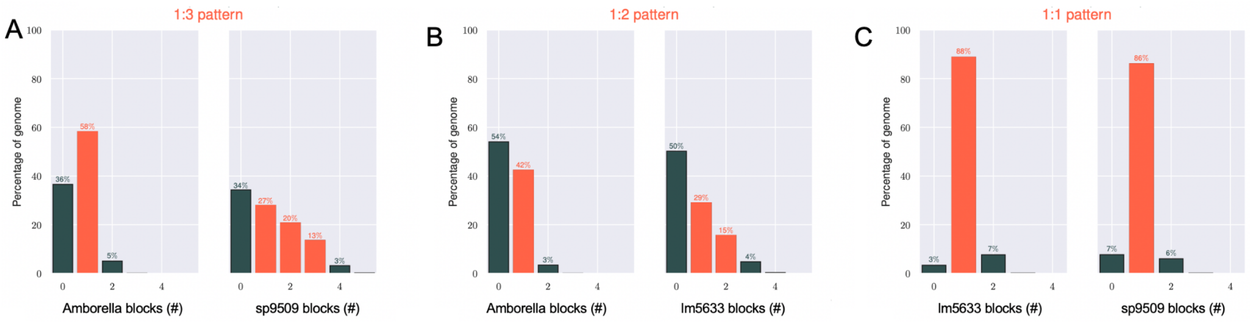

Supplemental Figure S4. Syntenic depth between Im5633, sp9509 and the basal plant Amborella. Genomes were aligned based on all the predicted protein coding sequence for Im5633 (22,873), sp9509 (18,507) and *A. trichopoda* (26,846). Syntenic blocks were identified with MCscan python version allowing a 20 gene window and requiring at least 5 syntenic gene pairs. Syntenic depth is on the x-axis and percent of the genome at that syntenic depth on the y-axis; syntenic depth highlighted in red. A) 1:3 syntenic depth between Amborella (*A. trichopoda*) and sp9509; B) 1:2 syntenic depth between Amborella (*A. trichopoda*) and Im5633; C) 1:1 syntenic depth between Im5633 and sp9509.

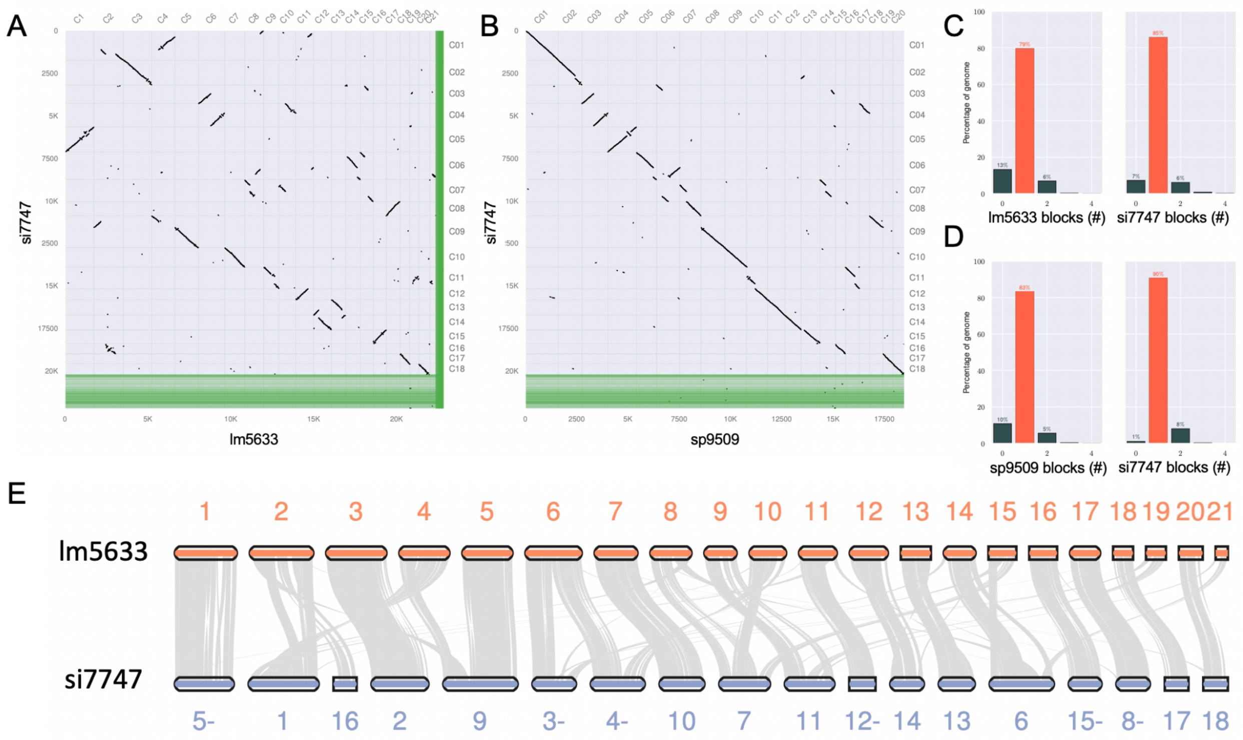

Supplemental Figure S5. The *Spirodela intermedia* genome (si7747) is syntenic with lm5633. Genomes were aligned based on all the predicted protein coding sequence for sp9509 (18,507), si7747 (22,245) and lm5633 (22,873). Syntenic blocks were identified with MCscan python version allowing a 20 gene window and requiring at least 5 syntenic gene pairs. A) Dotplot comparing si7747 and lm5633; green areas are smaller unplaced contigs. B) Dotplot comparing si7747 and sp9509; green areas are smaller unplaced contigs. C) 1:1 syntenic depth between lm5633 and si7747; D) 1:1 syntenic depth between sp9509 and si7747. Syntenic depth is on the x-axis and percent of the genome at that syntenic depth on the y-axis; syntenic depth highlighted in red. E) lm5633 chromosomes aligned to si7747 chromosomes with syntenic blocks (grey lines) anchoring positions between the two genomes. Chromosomes are the correct ratio between one another but are not to scale between the two species. A minus sign after the number means the chromosome has been flipped for visualization purposes.

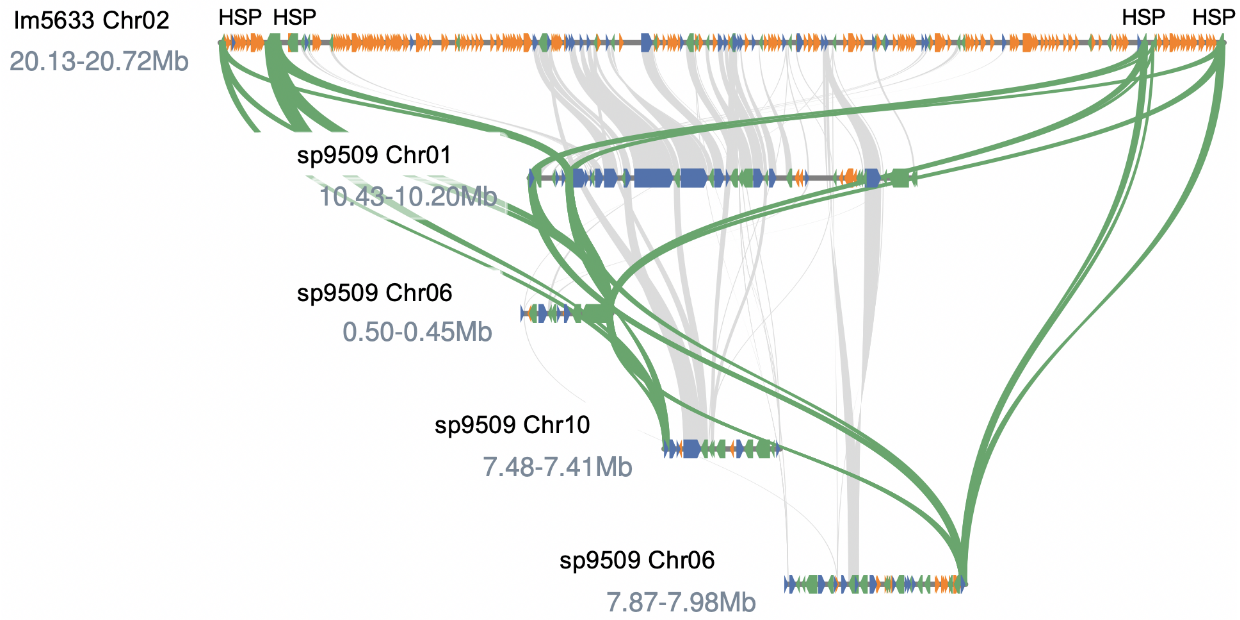

Supplemental Figure S6. Some regions of the sp9509 genome retain a 4:1 syntenic ratio with lm5633. *The HEAT SHOCK PROTEIN* (*HSP*) gene (green lines) is amplified by a tandem duplication (TD) and proximal duplication (PD) on Chromosome 2 (Chr02) in the lm5633 genome, while the region is retained in a 4:1 syntenic depth in sp9509. Genes are in blue (positive strand) and green (reverse strand), and transposable element fragments are in orange. Syntenic gene connections are grey lines connecting genome regions; green lines are specific to the HSP TD and PD copies.

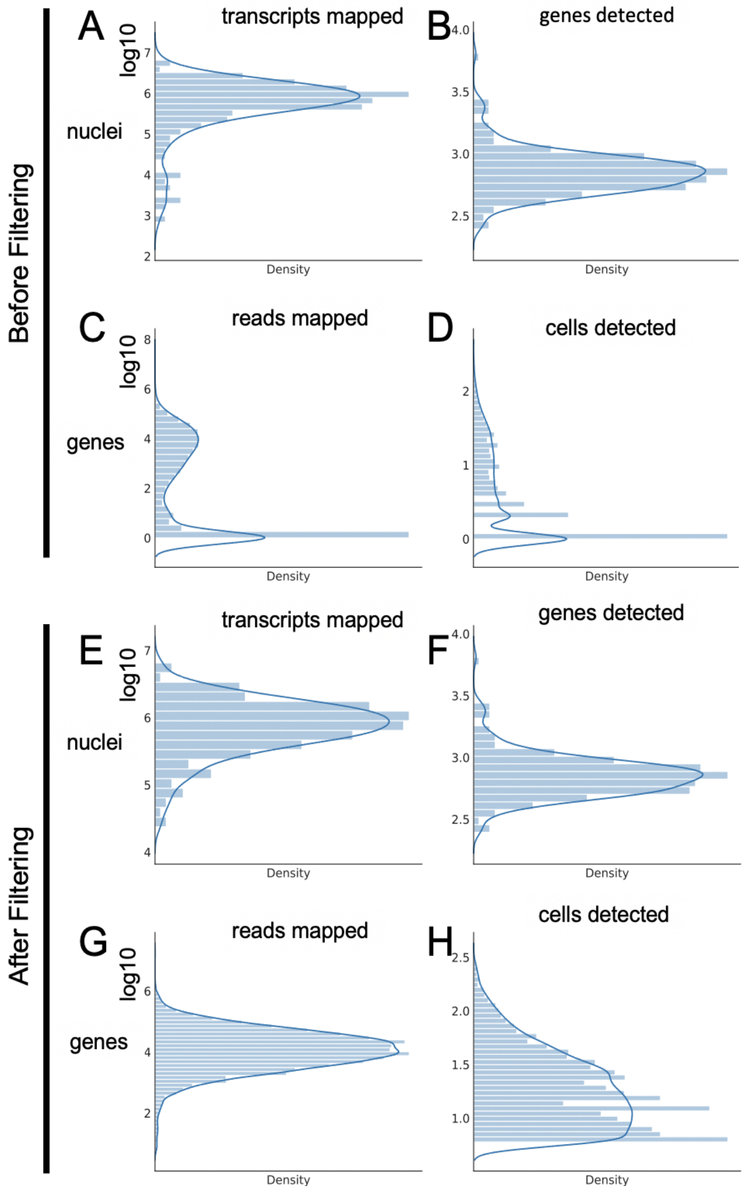

Supplemental Figure S7. Quality control of raw and post quality filtering of the expression matrix. A) Histogram describing the number of transcripts mapped per nucleus in log10. B) Histogram of the number of genes expressed within each nucleus. C) A histogram describing the number of transcripts mapped per gene. D) A histogram describing the number of cells expressing each gene. E) Following quality filtering for number of cells, number of mapped reads and number of expressed genes described in the methods. Histogram describing the number of transcripts mapped per nucleus in log10. F) Following quality filtering, a histogram describing the number of genes expressed within each nucleus G) Following quality filtering, a histogram describing the number of transcripts mapped per gene. H) Following QC filtering, a histogram describing the number of cells expressing each gene.

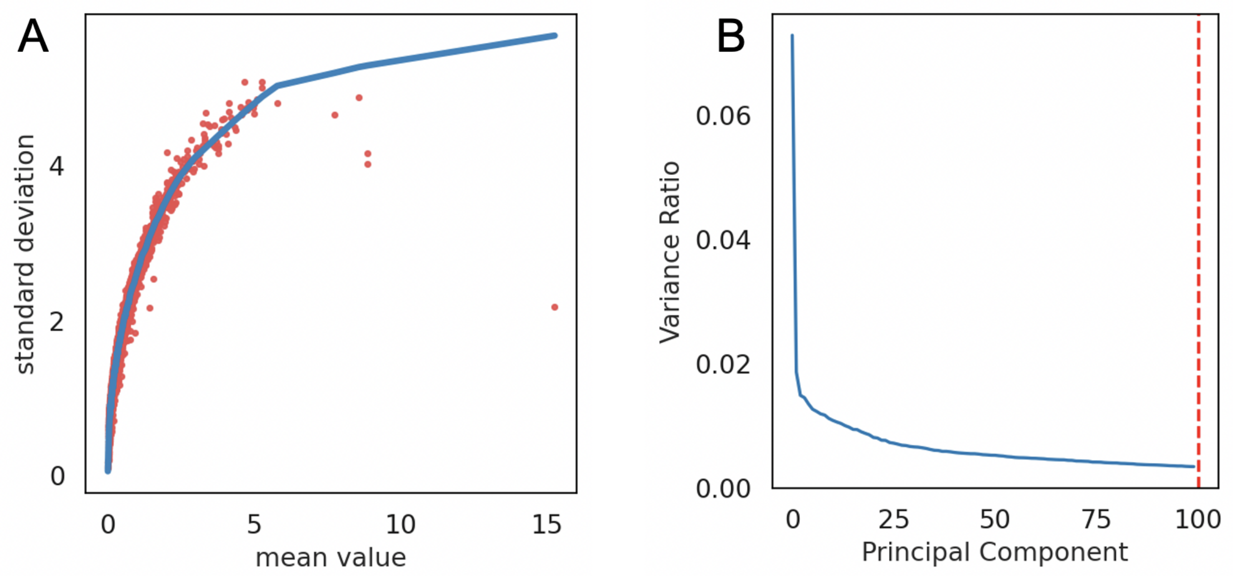

Supplemental Figure S8. Quality control of gene variance and PCA analysis. A) Following quality filtering, 8457 genes were determined to be variable genes with command stream.select_variable_genes(adata,loess_frac=0.01,n_genes=20000). Generally, only the most variable genes are used for downstream analysis. However, here we used all genes (red) spanning low and high expression variability as determined by expression standard deviation. B) The top 100 principal components were used for clustering.

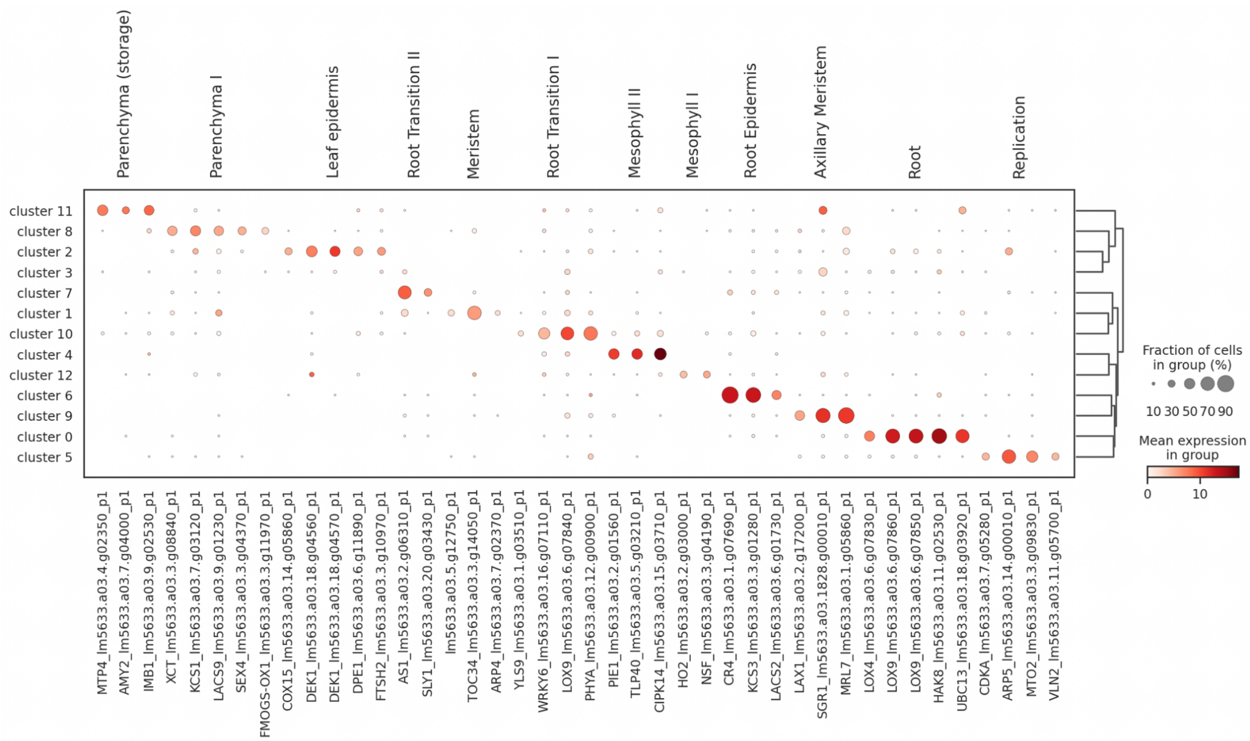

Supplemental Figure S9. Cell expression profiles of marker genes for identifying cell types. These marker genes were described in text for predicted cell type of each cluster. The size of each dot represents the percent of cells expressing the marker gene within each cluster and the color represents the transcript abundance from low (white) to high (red to black). These data show the uniqueness of each marker gene for each cluster.

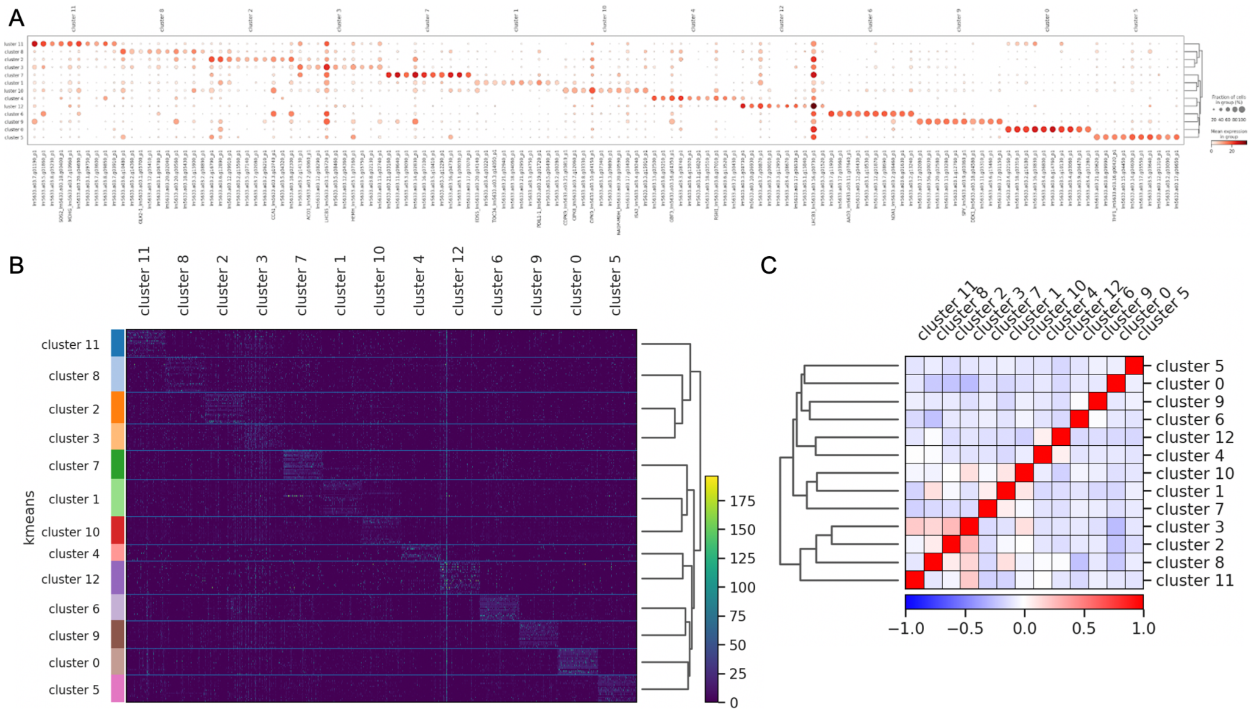

Supplemental Figure S10. The most significant marker genes for each cluster are highly specific. A) As described in the methods, the 10 most significant (p<0.01) marker genes per cluster from the STREAM analysis are plotted where the size of each dot represents the percent of cells expressing the marker gene within each cluster and the color represents the transcript abundance from low (white) to high (red to black). These data show the uniqueness of each marker gene for each cluster. Some genes (e.g. *LHCB3*) are expressed in all clusters but is considered a marker gene based on significantly greater expression in a specific cluster. Marker genes for each cluster can be found in Supplemental Table S9. B) A heatmap of the top 100 more significant marker genes per cluster is presented. C) Pearson correlation based on expression profiles for each cluster showing uniqueness between clusters. These data describe the unique expression profiles for each cluster as low Pearson correlation (blue) between clusters is most common.
